# Antioxidant Properties of Kynurenines: Density Functional Theory Calculations

**DOI:** 10.1371/journal.pcbi.1005213

**Published:** 2016-11-18

**Authors:** Aleksandr V. Zhuravlev, Gennady A. Zakharov, Boris F. Shchegolev, Elena V. Savvateeva-Popova

**Affiliations:** 1 Neurogenetics Laboratory, Pavlov Institute of Physiology RAS, St. Petersburg, Russia; 2 Department of Experimental Physiology and Pharmacology, V. Almazov Federal Heart, Blood and Endocrinology Centre, St. Petersburg, Russia; University of Houston, UNITED STATES

## Abstract

Kynurenines, the main products of tryptophan catabolism, possess both prooxidant and anioxidant effects. Having multiple neuroactive properties, kynurenines are implicated in the development of neurological and cognitive disorders, such as Alzheimer's, Parkinson's, and Huntington's diseases. Autoxidation of 3-hydroxykynurenine (3HOK) and its derivatives, 3-hydroxyanthranilic acid (3HAA) and xanthommatin (XAN), leads to the hyperproduction of reactive oxygen species (ROS) which damage cell structures. At the same time, 3HOK and 3HAA have been shown to be powerful ROS scavengers. Their ability to quench free radicals is believed to result from the presence of the aromatic hydroxyl group which is able to easily abstract an electron and H-atom. In this study, the redox properties for kynurenines and several natural and synthetic antioxidants have been calculated at different levels of density functional theory in the gas phase and water solution. Hydroxyl bond dissociation enthalpy (BDE) and ionization potential (IP) for 3HOK and 3HAA appear to be lower than for xanthurenic acid (XAA), several phenolic antioxidants, and ascorbic acid. BDE and IP for the compounds with aromatic hydroxyl group are lower than for their precursors without hydroxyl group. The reaction rate for H donation to *O-atom of phenoxyl radical (Ph-O*) and methyl peroxy radical (Met-OO*) decreases in the following rankings: 3HOK ~ 3HAA > XAA_OXO_ > XAA_ENOL_. The enthalpy absolute value for Met-OO* addition to the aromatic ring of the antioxidant radical increases in the following rankings: 3HAA* < 3HOK* < XAA_OXO_* < XAA_ENOL_*. Thus, the high free radical scavenging activity of 3HAA and 3HOK can be explained by the easiness of H-atom abstraction and transfer to O-atom of the free radical, rather than by Met-OO* addition to the kynurenine radical.

## Author Summary

Kynurenines, the tryptophan metabolites with multiple biological activities, regulate the production of reactive oxygen species (ROS) during several neurodegenerative diseases. Many experiments show that kynurenines can be both prooxidants and antioxidants depending on their concentration, mode of action, and cell redox potential. However, there is lack of computational studies of kynurenines properties which could help us better understand the biophysical mechanism of their antioxidant activity. We performed the computations of kynurenines' hydrogen and electron donating power, both in the gas phase and in water solution. We found that aromatic hydroxyl group facilitates hydrogen and electron abstraction by kynurenines, in agreement with experimental data and computations earlier performed for phenolic antioxidants. We revealed the correlations of kynurenines' antioxidant power with their electronic structure, charge, and surroundings. We also found that 3-hydroxykynurenine and 3-hydroxyanthranilic acid can fastly quench free radicals by hydrogen atom donation. Hence both of them are potent antioxidants. The therapeutic strategy may be to inhibit their oxidative dimerization leading to ROS production.

## Introduction

**Fig 1:**
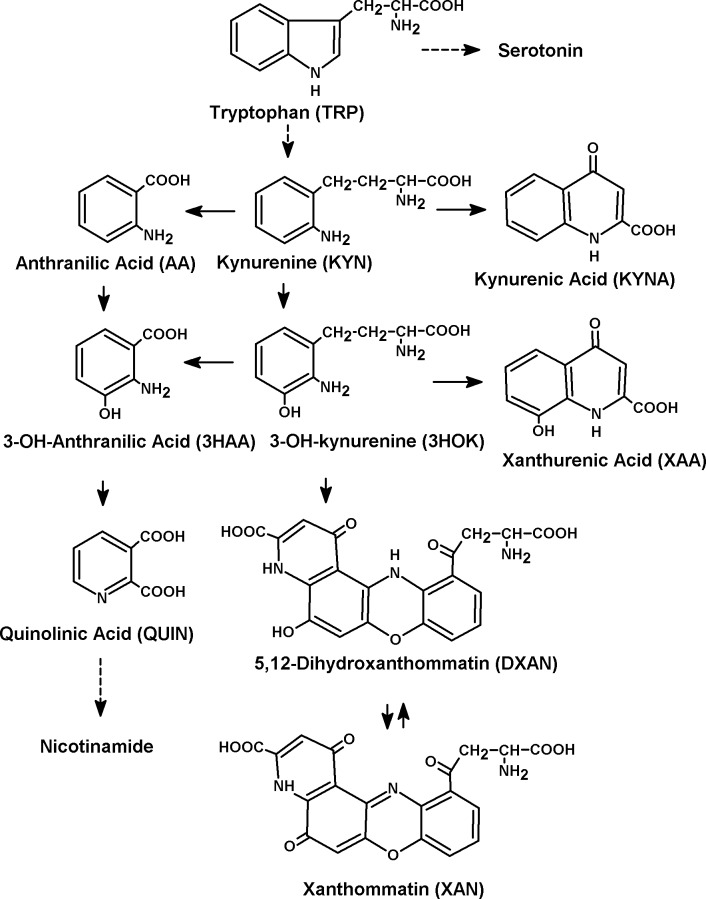
Kynurenine pathway of tryptophan methabolism. The implication of KP in a variety of physiological and pathophysiological processes, including anti-microbial and anti-tumor defense, neuropathology, immunoregulation, and antioxidant activity, has been ever drawing attention to biochemical properties of kynurenines [1]. Kynurenines are considered to be involved in ageing and numerous neurodegenerative diseases, such as Alzheimer's disease (AD), Parkinson's disease (PD), Huntington's disease (HD), amyotrophic lateral sclerosis (ALS), etc. [2–4].

The kynurenine pathway (KP), the primary route of tryptophan degradation in mammalian cells, includes kynurenine (KYN), kynurenic acid (KYNA), 3-hydroxykynurenine (3HOK), 3-hydroxyanthranilic acid (3HAA), quinolinic acid (QUIN), and other metabolites collectively called kynurenines (Fig 1).

There are multiple mechanisms of kynurenines' action on nervous system. QUIN and KYNA, the ligands of ionotropic glutamate receptors [5,6], modulate neurodegenerative processes in the brain [7]. The autoxidation of 3HOK and 3HAA leads to the hyperproduction of reactive oxygen species (ROS) which damage cellular lipids, proteins, and DNA [8–10]. Kynurenine 3-monooxygenase (KMO), an enzyme producing 3HOK from KYN, has been linked to the pathophysiology of HD by a mechanism involving ROS [11]. Accumulation of 3HOK in the central nervous system of Drosophila *cardinal* mutant leads to the progressive memory loss [12]. Since 3HOK is capable of auto-condensation, the eyes of this mutant, as well as the color of mammalian lens cataract [13] progressively get brown on ageing. The Drosophila eye color mutants are started to be envisioned as a therapeutic tools for HD [14].

At the same time, both 3HOK and 3HAA were shown to be powerful antioxidants scavenging peroxyl radicals [15,16]. Xanthurenic acid (XAA), a product of KYNA hydroxylation, has similar antioxidative properties, but its rate of interaction with free radicals is slower [15]. Tryptophan and its catabolites without aromatic hydroxyl group, such as kynurenine (KYN), KYNA, and anthranilic acid (AA) have no effect on peroxy-mediated oxidation. Thus, phenolic hydroxyl group is important for antioxidant activity of kynurenines. Antioxidants are supposed to beneficially interfere with diseases-related oxidative stress, however, the interplay of endogenous and exogenous antioxidants with the overall redox system is far from clear [17].

Phenolic compounds suppress lipid peroxidation due to their ability to react with free radicals at a faster rate than with the substrate [18,19]. There are two main pathways of phenolic antioxidants quenching free radicals: electron transfer and H-atom transfer. H-atom easily abstracted from the aromatic OH-group interacts with peroxyl radical ROO* produced during lipid peroxidation and breaks the chain reaction:

Ar−OH+ROO*→Ar−O*+ROOH (1)

There are two pathways of hydrogen transfer: hydrogen atom transfer (HAT) and proton-coupled electron transfer (PCET) [20]. HAT is preferable when electron density of singly occupied molecular orbital (SOMO) in the transition structure (TS) lies along the same line as the O…H…O bond and H is transferred between the oxygens as a whole particle. PCET is preferable when SOMO is orthogonal to O…H…O bond, as in phenoxyl-phenol complex, and proton is transferred between oxygen σ lone pairs forming hydrogen bonds with them, while the electron is transferred between oxygen π-orbitals.

Also, phenolic antioxidant radicals are able to quench peroxyl radical via its addition to the aromatic ring at ortho- or para-position. In order to trap the radical and not to react with hydrocarbon R-H substrate, an antioxidant should have less value for the homolytic O-H bond dissociation enthalpy (BDE) than ROO-H and R-H. Moreover, antioxidant radical should be kinetically stable to prevent its reaction with substrate [21,19]. Thus, the antioxidant power is not an absolute property of Ar-OH, but depends on the substrate which should be protected.

The toxicity of 3HOK depends mainly on the products of its oxidative dimerization, such as hydrogen peroxide, xanthommatin (XAN), 4,6-dihydroxyquinolinequinonecarboxylic acid (DHQCA), their active free radical forms, and *o-*aminoquinone [22]. Ommochromes XAN and dihydroxanthommatine (DXAN), the brown eye pigments, easily transform into each other under physiological conditions [23,24]. DXAN is synthesized from 3HOK by phenoxazinone synthetase (PHS)–the process disturbed by the *cardinal* mutation [23]. PHS catalyzes two consecutive abstractions of H-atoms from the hydroxyl group of o-aminophenols, 3HOK or 3HAA, followed by their non-enzymatic condensation to phenoxazinone [25]. The formation of ommochromes can also result from non-enzymatic oxidation of 3HOK [26]. High concentration of 3HOK catabolite hydrogen peroxide induces apoptotic cell death in neuronal cell cultures [27]. 3HOK and 3HAA generate superoxide anion and hydrogen peroxide in the presence of copper–the process leading to the formation of a quinoneimine structure [28]. Both amino and hydroxyl aromatic groups are important for lowering 3HOK and 3HAA oxidation potential. Initially, they can be two-electron donors with antioxidant activity, but their quinoneimine products are highly reactive and damage cell structures. Pro- and antioxidant power of o-aminophenols depends on the whole activity of the redox systems in cell [29].

Other kynurenine metabolites also possess pro- and antioxidant activity [4]. In particular, KYNA is able to scavenge hydroxyl radicals, superoxide anion radicals, and peroxynitrite, decreasing lipid peroxidation and ROS formation [30]. QUIN affects the ROS level only together with iron ions; the pro- and antioxidant effects of QUIN are concentration-dependent [31]. Free radicals scavenging mechanisms shown for non-o-aminophenol kynurenines include electron transfer, metal ion chelation, destruction of carbon skeleton, and radical addition to the aromatic ring [4].

Whereas experimental data regarding chemical and physiological properties of kynurenines are abundant and diverse, there are few computational studies on kynurenines. Quantum chemical calculations could provide a better understanding of the mechanisms of kynurenines' antioxidant activity. In this study, the redox properties of kynurenines and several synthetic phenolic antioxidants were investigated computationally using density functional theory (DFT) approach. The validity of B3LYP methods to model phenolic antioxidants and free radical reactions has already been proved [18,32]. The methodology was similar to that of [18,33]: the energies of frontier highest occupied and lowest unoccupied molecular orbitals (E_HOMO_, E_LUMO_), phenolic O-H bond dissociation enthalpy (BDE), and ionization potential (IP) were calculated and compared for structures fully optimized in the gas phase. We also studied the influence of water solvation on the chemical properties of antioxidants. Finally, we modeled the kinetic behavior of hydroxykynurenines interaction with phenoxyl and peroxyl radicals.

## Results

### Phenolic antioxidants and hydroxykynurenines; BDE, IP, and frontier orbital energies

Optimal geometries for kynurenines and synthetic antioxidants with substituted phenolic groups were calculated at different levels (Table 1). Six compounds with experimentally known BDE values are used as standards for the estimation of the validity of computational methods. Despite high diversity of chemical structures, Pearson correlation coefficient R is high for level II (0.870 and 0.867 for BDE and BDE_COR_, respectively; p<0.05) and III (0.865, 0.863; p<0.05), being less for level I (S1 Table) (0.717, 0.710; p > 0.1). Total spin <S2> shows small spin contamination ranging from 0.75 to 0.80 for all free radicals (II, III), being abnormally high for some radicals calculated at level I. Thus, (I) computational data were omitted from further analysis. BDE and BDE_COR_ (II, III) for phenol are greatly higher than the experimental value and the value previously calculated at the same level of theory; the cause is explained in Methods section. With the exception of phenol, the correlation of BDE/BDE_COR_ with experimental values is very strong (R = 0.959 and 0.974 for level II and III, respectively; p < 0.05, n = 5). The goal of this study was not the precise calculations of energy values, but rather the comparison of such values for different antioxidants. Thus, DFT calculations at level II or III can be used to predict the relative antioxidant power of the studied compounds.

**Table 1:** Hydrogen donating ability of kynurenines and phenolic antioxidants (levels II-III, III(LC-BLYP)).

	II					III					III (LC- BLYP)					BDE_EXP_
Compound	E_HOMO_	E_LUMO_	H-L gap	BDE	BDE_COR_	E_HOMO_	E_LUMO_	H-L gap	BDE	BDE_COR_	E_HOMO_	E_LUMO_	H-L gap	IP	μ opt	
Water	-182.731	39.282	-222.013	116.232	109.333	-188.064	17.445	-205.509	119.686	112.784						118.8^b^
Methane	-244.101	73.983	-318.084	112.837	104.838	-247.803	32.505	-280.308	111.605	103.813						105.0^b^
Phenol	-137.424	0.816	-138.240	106.394	99.481	-143.449	-7.279	-136.169	110.219^a^	103.212						88.7^b^
L-3HOK_NH3+_	-223.581	-124.937	-98.644	90.534	83.450	-228.162	-128.577	-99.586	93.882	86.760						
ASC	-143.574	-19.578	-123.996	83.468	77.327	-148.720	-23.406	-125.314	86.743	80.493						81.0^c^
DIBP	-132.216	2.761	-134.977	81.081	74.391	-138.428	-5.020	-133.408	84.705	77.898						
DIBA	-133.910	-35.894	-98.017	79.911	73.135	-139.307	-41.604	-97.703	83.253	76.395						
XAA_OXO_	-134.036	-45.494	-88.542	77.841	71.339	-139.997	-50.703	-89.295	81.288	74.746	-174.574	-12.431	-162.143	177.179	0.2063	
DTBP	-131.400	5.522	-136.922	77.797	69.930	-137.783	-2.008	-135.730	81.480	74.608	-166.017	28.699	-194.715	173.437	0.1627	82.8^d^
DTBA	-133.032	-35.266	-97.766	76.615	69.733	-138.805	-41.102	-97.703	79.988	73.097						
L-3HOK	-121.235	-28.803	-92.432	73.880	67.572	-126.506	-33.885	-92.620	77.190	71.473	-159.200	2.061	-161.261	160.589	0.1907	
2-NH_2_-Phenol	-117.909	9.601	-127.510	74.374	67.849	-123.306	1.883	-125.188	77.565	70.970	-158.709	40.401	-199.110	161.444	0.2052	81.3^e^
D-3HOK	-121.235	-28.803	-92.432	73.882	67.623	-126.506	-33.948	-92.558	77.197	70.932						
3HAA	-121.172	-22.026	-99.146	73.846	67.569	-126.506	-27.548	-98.958	77.193	70.896	-162.369	11.439	-173.808	164.887	0.2098	
XAA_OXO/CO2-_	-39.972	53.652	-93.624	72.132	65.767	-48.318	46.310	-94.628	75.621	69.243						
DXAN	-106.551	-46.185	-60.366	64.973	58.867	-112.261	-51.142	-61.119	68.194	62.071						
3HAA_CO2—_	-22.716	90.424	-113.140	63.866	57.420	-30.936	82.266	-113.203	66.997	60.499						

II: B3LYP/6-31G(d); III: B3LYP/6-311G(d,p); III (LC-BLYP): B3LYP/6-311G(d,p) full geometry optimization, LC-BLYP single point energy. All values are in kcal/mol. Abbreviations: BDE_EXP_−experimental BDE values, CO_2_^-^ –ionized carboxyl group, NH_3_^+^–ionized aromatic amino group, μ opt–the optimal values of range-separation parameter μ. The rows in table are arranged in accordance with BDE_COR_ (III) values. References: a. Gaussian 98 value of BDE is 93.129 kcal/mol; b–[34], c–[35], d–[36], e–[37].

The values of E_HOMO_, E_LUMO_, BDE, and BDE_COR_ at level II are highly correlated with those calculated at level III (R = 0.98–1.00) In general, level III gives a slightly higher BDE/BDE_COR_ than level II. BDE/BDE_COR_ calculated at B3LYP and HCTH/407 levels of DFT (basis set II) are strongly correlated (R = 0.999 and 0.998, respectively; p < 0.05, n = 16, without phenol). HCTH/407 gives slightly lower values of BDE than B3LYP (S1 Table; ΔE = 2.252±0.751 kcal/mol). For phenol, BDE_HCTH/407_ is 84.702 kcal/mol, which is much closer to the experimental value. Thus, both functionals can be used to estimate BDEs for kynurenines and phenolic antioxidants.

The rankings for O-H homolytical BDE_COR_ are nearly the same at levels II and III. O-H bond is the strongest in water and the weakest in negatively charged 3HAA_CO2_-. BDE_COR_ values for 3HOK and 3HAA are close to that for 2-aminophenol, their structural precursor. 2-aminophenol is an antioxidant with a large decrease in the O-H BDE compared to phenol [38]. L-3HOK and D-3HOK optical isomers have almost equal BDE values. 3HOK and 3HAA are characterized by the decreased energies of H abstraction compared to phenol and its derivatives DIBP and DTBP, both native and modified by propenoic acid (DIBA, DTBA). Total energy for XAA oxo form is lower by 7.4 kcal/mol than for enol form (level III); therefore, we used the oxo form in the majority of calculations. XAA_OXO_ is close to phenolic antioxidants in its H donating properties.

B3LYP, as well as most DFT methods, is known to give E_HOMO_ and E_LUMO_ in a very poor agreement with experiment, significantly underestimating H-L gap. Using of tuned range-separated hybrid functionals can solve this problem [39,40]. We have computed E_HOMO_, E_LUMO_, H-L gap, and IP for five compounds optimized at level III, B3LYP (L-3HOK, 3-HAA, XAA_OXO_, 2-aminophenol, and DTBP) using tuned LC-BLYP range-separated functional. Indeed, LC-BLYP gives significantly higher absolute values for H-L gap (ΔE_LC-BLYP–B3LYP_ = -69.849±8.135 kcal/mol), and IP values are close to -E_HOMO_ (ΔE_-HOMO–IP_ = -3.333 ±2.929 kcal/mol). The optimal range-separation parameter μ values are close for four aminophenols (~0.20) and differ from that for phenolic antioxidant DTBP. At the same time, E_HOMO_, E_LUMO_, and H-L gap values calculated by III(LC-BLYP) and III(B3LYP) are highly correlated (R = 0.911, 0.989 and 0.955, respectively), thereby the rankings for electron donating power are virtually the same in both cases. XAA_OXO_ has the highest values for IP and -E_HOMO_, whereas L-3HOK is the best electron donor among the uncharged hydroxykynurenines.

There is a moderate negative correlation between E_HOMO_ and BDE/BDE_COR_ (levels II, III) (S2 Table). The correlation between H-L gap and BDE/BDE_COR_ is even stronger. Hence the ability of O-H bond homolytical dissociation tends to increase along with the lowering of E_HOMO_ and H-L gap absolute values. The correlation between E_LUMO_ and BDE/BDE_COR_ is not significant at all levels. For the compounds with an ionized group, such as 3HAA_CO2-_, XAA_CO2-_, and L3HK_NH3+_, E_HOMO_ significantly differ from those of uncharged compounds. 3HAA_CO2_- and XAA_OXO/CO2-_ are more powerful H donors than the uncharged forms. On the contrary, protonation of NH_2_ group in L-3HOK phenolic ring significantly complicates O-H dissociation. This is in agreement with the fact that electron-donating groups reduce O-H BDE, thus enhancing antioxidant activity, whereas electron-withdrawing substitutions raise it [41,42,18]. DIBP and DTBP, the substances with skeletal isomerism, have similar BDEs. However, for each of them, BDE is closer to that of its propenoic derivative than to BDE of its isomer. Hence the side chain isomerism significantly affects the H- donating properties of phenolic group. DXAN has the least stable O-H bond among the uncharged compounds, making it a potent anioxidant with the high H donating ability.

In order to check the possible effect of the basis set superposition error (BSSE) on BDE, BSSE correction was performed for phenol, DIBP, and DXAN with a small, intermediate, and large hydrocarbon moiety of a radical. The values of BSSE (III) are -0.722, -0.832 and -1.45 kcal/mol, respectively, being small and similar in all compounds. The decrease of BDE for bulky antioxidants cannot be explained by the growth of BSSE.

### Kynurenines and phenolic antioxidants; frontier molecular orbitals and spin-orbits

**Fig 2:**
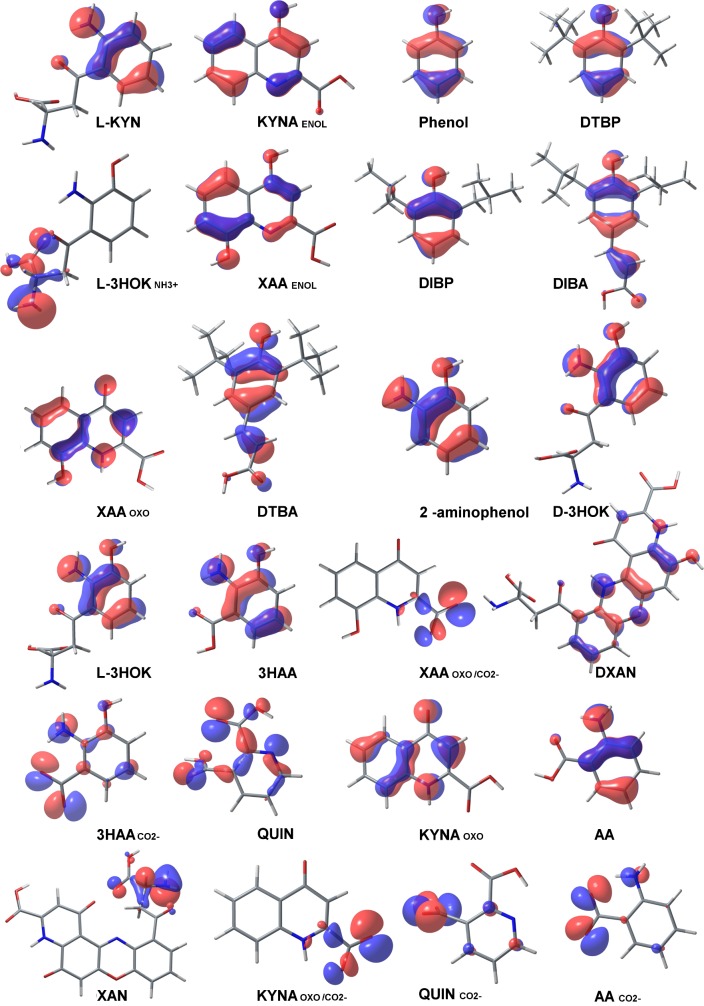
The highest occupied molecular orbitals (HOMOs) of kynurenines and phenolic antioxidants. **Color scheme, atoms: H–white, C–grey, O–red, N–blue. Isosurface value: 0.05.** The lowest unoccupied molecular orbital (LUMO) has multiple nodes in the bonding region, mainly localized outside of the OH group (S1 Fig). For the ionized compounds, LUMO is moved from the charged group to the aromatic system; for L-3HOK_NH3+_, its form is nearly the same as that of L-3HOK HOMO. The geometry of HOMOs closely resembles the geometry of spin-orbits of cation radicals (S2 Fig). Hence HOMO correctly reproduces the geometry of the electron density in phenolic cations calculated at the DFT level. The main differences are between anion HOMOs and the corresponding spin-orbits after electron abstraction: spin-orbit is localized mainly on aromatic atoms and partly on α-carboxylic group. Spin-orbit of radicals after H-atom abstraction is localized on O*-atom and π-conjugated moiety, including the aromatic system and unsaturated side chains (S3 Fig). Delocalization is low in L-KYN, KYNA_ENOL_, and phenol radicals. This may explain their lower capability to donate H compared with hydroxykynurenines and substituted phenols.

The geometry of frontier molecular orbitals and spin-orbits was calculated at level III for hydroxykynurenines and their precursors, as well as for their derivatives without an aromatic hydroxyl group. The highest occupied molecular orbital (HOMO) of phenolic antioxidants and kynurenines is localized mainly on the phenolic ring. HOMO is divided into two parts: the first part occupies phenolic OH group and three approximate C atoms, and the second part occupies the opposite two or three C atoms (Fig 2). HOMO also occupies unsaturated and polar groups attached to the phenolic ring, such as the aromatic NH_2_ group of L-3HOK and 3HAA, which HOMO's and spin-orbit's geometry is virtually the same as that for 2-aminophenol. Together with the aromatic rings, OH and NH_2_ groups form a π-conjugated systems known to decrease IP [43]. DXAN has the largest conjugated system allocated mainly to phenoxazinone structure, which possibly facilitates H-atom and electron abstraction. For the ionized compounds, HOMO is moved from the aromatic hydroxyl group to the charged group.

### H-atom and electron donating power of kynurenines and phenolic antioxidants in the gas phase and water solution

**Fig 3:**
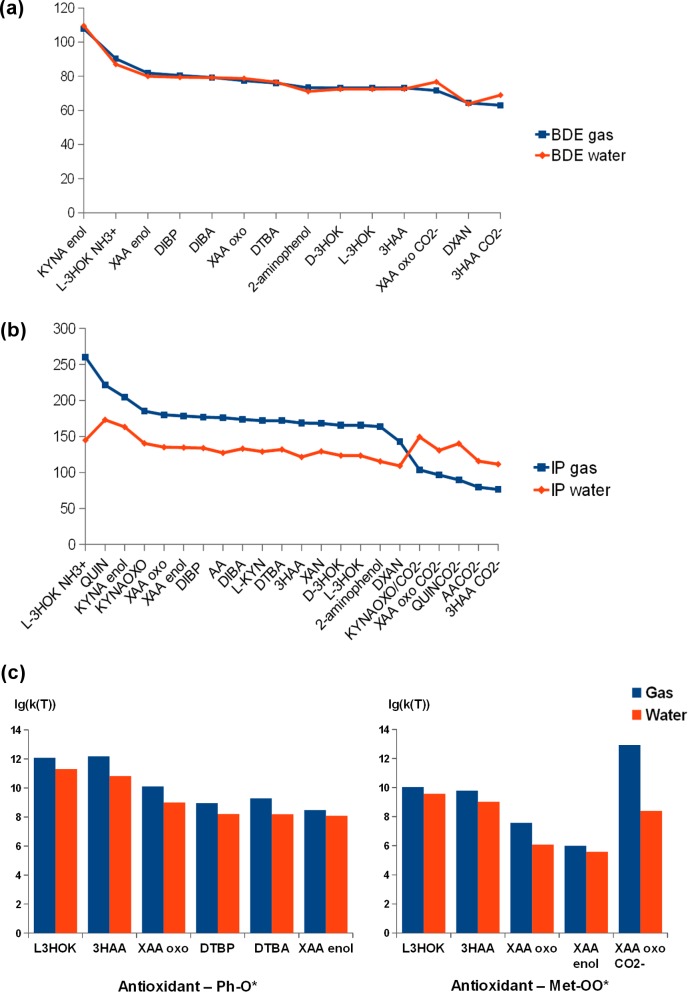
BDE, IP, and k(T) values of kynurenines in the gas phase and water solution. **(a) BDE values. (b) IP values. (c) k(T) values (in logarithmic form). Blue–the gas phase, red–water solution.** BDE (IV) rankings for H-atom in the gas phase are nearly the same as for BDE/BDE_***COR***_ (II, III). BDE is abnormally high for two symmetric compounds, phenol and DTBP. DTBA have much lower BDE, which is in agreement with its high antioxidant power. BDE for DIBP and DIBA are close to those for DTBA and, presumably, the true BDE value for DTBP. For the uncharged kynurenines with the OH group, BDE is maximal for KYNA_***ENOL***_ and minimal for DXAN, both in the gas phase and in water solution. XAA and KYNA have smaller BDE in oxo form than in enol form.

BDE, IP and frontier orbital energies for compounds optimized at level III were calculated at level IV (B3LYP/6-311+(O)+G(d)) in the gas phase and water solution (Table 2, Fig 3A and 3B). A moderate negative correlation between BDE and E_HOMO_/H-L gap values was observed, as well as for levels I-III, both in the gas phase and in water solution (S2 Table, second part). A strong correlation between adaibatic IP and -E_HOMO_ or the so called vertical IP can be seen. This is in accordance with Koopmans' theorem, applicable in high approximation for outer valence Kohn-Sham orbitals [44]. The difference between IP and -E_HOMO_ is 36.7±3.3 kcal/mol in the gas phase, in agreement with the fact that B3LYP underestimates the absolute values for E_HOMO_ [39]. In water solution, IP becomes slightly lower than the negative of E_HOMO_. BDE is positively correlated with IP; thereby the electron and H donating capacities of the studied compounds are interrelated.

**Table 2:** Hydrogen and electron donating abilities of kynurenines and phenolic antioxidants in the gas phase and water solution.

	Gas phase								Water solution							
Compound	BDE	IP	E_HOMO_	E_LUMO_	H-L gap	δ_SD_(R*)	SD(O*)	SD(C_P_)	BDE	IP	E_HOMO_	E_LUMO_	H-L gap	δ_SD_ (R*)	SD(O*)	SD(C_P_)
KYNA_ENOL_	107.738	204.495	-151.606	-49.448	-102.158	0.201	0.900		109.569	163.163	-150.288	-57.543	-92.746	0.203	0.908	
Phenol	**106.511**	190.455	-144.829	-7.467	-137.362	0.266	0.925	**-0.009**	**107.652**	138.876	-144.829	-14.307	-130.522	0.264	0.919	**-0.010**
DTBP	**104.459**	174.788	-138.366	-5.773	-132.593	0.128	0.761	**-0.009**	**104.919**	132.524	-140.123	-10.228	-129.894	0.125	0.748	**-0.010**
L-3HOK_NH3+_	90.276	260.317	-230.610	-131.024	-99.586	0.137	0.404	0.397	86.990	144.782	-160.015	-59.362	-100.652	0.137	0.378	0.415
XAA_ENOL_	81.829	178.406	-141.880	-47.063	-94.817	0.159	0.314	0.455	79.959	134.545	-141.190	-56.162	-85.027	0.153	0.283	0.460
DIBP	80.442	176.823	-139.621	-8.660	-130.961	0.118	0.377	0.384	79.385	133.891	-141.817	-13.491	-128.326	0.116	0.353	0.395
DIBA	79.279	173.657	-141.817	-44.616	-97.202	0.108	0.301	0.358	79.184	132.967	-141.441	-53.903	-87.538	0.109	0.285	0.363
XAA_OXO_	77.416	180.001	-142.758	-55.158	-87.600	0.132	0.316	0.391	78.693	135.095	-141.441	-61.872	-79.568	0.132	0.299	0.404
DTBA	76.004	171.908	-140.625	-43.800	-96.825	0.108	0.281	0.368	76.570	131.893	-139.935	-52.648	-87.287	0.110	0.274	0.373
2-aminophenol	73.240	163.623	-124.310	-7.216	-117.093	0.125	0.299	0.266	71.045	115.325	-125.878	-6.589	-119.289	0.111	0.258	0.228
D-3HOK	73.172	165.467	-128.702	-36.584	-92.118	0.098	0.292	0.316	72.432	123.471	-128.075	-43.863	-84.212	0.093	0.264	0.306
L-3HOK	73.166	165.428	-128.702	-36.584	-92.118	0.098	0.292	0.316	72.420	123.372	-128.075	-43.926	-84.149	0.093	0.264	0.306
3HAA	73.131	168.620	-128.953	-31.187	-97.766	0.121	0.296	0.320	72.548	121.435	-128.827	-39.031	-89.797	0.114	0.267	0.310
XAA_OXO/CO2-_	71.619	96.421	-59.300	37.337	-96.636	0.116	0.284	0.334	76.700	130.611	-135.103	-41.792	-93.311	0.127	0.290	0.381
DXAN	64.293	142.619	-114.771	-55.221	-59.551	0.062	0.189	0.254	63.834	109.081	-116.152	-62.814	-53.338	0.059	0.160	0.223
3HAA_CO2-_	62.992	*76*.*302*	-37.964	49.071	-87.035	0.096	0.260	0.166	68.937	*111*.*423*	-121.674	-16.503	-105.171	0.099	0.249	0.198
QUIN		221.492	-182.229	-57.103	-125.125					173.091	-180.534	-58.986	-121.548			
KYNA_OXO_		185.212	-146.586	-57.731	-88.855					140.357	-145.654	-62.500	-83.145			
AA		176.032	-134.475	-32.568	-101.907					127.162	-133.408	-40.223	-93.185			
L-KYN		171.962	-133.910	-37.713	-96.197					128.916	-132.342	-44.490	-87.851			
XAN		168.143	-159.952	-88.918	-71.034					129.194	-152.171	-87.035	-65.135			
KYNA_OXO/CO2-_		103.657	-61.433	36.709	-98.142					149.314	-138.052	-43.424	-94.628			
QUIN_CO2-_		89.647	-50.201	33.634	-83.835					140.080	-149.912	-50.640	-99.272			
AA_CO2-_		*79*.*421*	-39.784	64.633	-104.417					*115*.*706*	-124.686	-17.759	-106.928			

All values are in kcal/mol. Abbreviations: CO_2_^-^: ionized carboxylic group, NH_3_^+^: ionized amino group, ENOL, OXO: enol and oxo tautomers. The rows for the compounds with H-atom abstraction (upper part) are arranged in accordance with values for BDE in the gas phase, the rows in the lower part are arranged in accordance with values for IP in the gas phase. δ_SD_(R*)–the standard deviation of spin density on radical atoms after H abstraction, SD(O*)—spin density on radical O* atom, SD(C_P_)–spin density on radical C atom in para-position relative to O* atom. Bold: the values significantly different from experimental and previously calculated values. Italics: the radical structures were optimized in water solution.

BDE of aromatic antioxidants is strongly correlated with standard deviation of Mulliken spin density (δ_SD_) on radical (R_GAS_ = 0.879, R_WATER_ = 0.917; p < 0.05, n = 14) and spin density (SD) on radical O* atom (R_GAS_ = 0.910, R_WATER_ = 0.951; p = 0.05, n = 14) after H abstraction. BDEs for phenol and DTBP were not considered due to significant deviations from experimental values. Also, BDE is strongly correlated with SD on radical C_PARA_ aromatic atom after H abstraction (R_GAS_ = 0.849, R_WATER_ = 0.876; p < 0.05, n = 13, without KYNA_ENOL_ which has N instead C_PARA_).

There is no significant correlation between IP and δ_SD_ for kynurenine radicals after single electron abstraction. Thus, electron delocalization on kynurenines seems to be more important for H-atom donation activity than for the electron donation activity.

OH group bonded to the aromatic ring significantly increases the ability of kynurenines to donate H-atom and electron. L-KYN C_3_-H BDE is much higher than L-3HOK O_3_-H BDE: the difference is 40.4 kcal/mol in the gas phase and 40.3 kcal/mol in water solution (level V: B3LYP/6-311++(d,p)). IP value is lower for XAA_OXO_, L-3HOK, and 3HAA than for KYNA_OXO_, L-KYN, and AA, respectively (ΔIP_GAS_ = 6.4±2.9 kcal/mol, ΔIP_WATER_ = 5.5±0.6 kcal/mol). However, the relative IP rankings are the same for compounds with and without OH group: KYNA has higher IP compared to L-KYN and AA, as well as XAA compared to L-3HOK and 3HAA. This is in agreement with experimental data: the electrochemical potential E_pa_ for kynurenines has been shown to decrease in the following rankings: KYNA > KYN > AA > 3HOK > 3HAA [29]. There is a strong positive correlation between experimental E_pa_ and IP calculated for non-ionized compounds in water solution at level IV (R = 0.924; p < 0.05, n = 5). For compounds with an ionized CO_2_ group, the correlation is not significant (R = 0.804; p > 0.1, n = 5), probably, due to the small sampling. QUIN is the least powerful electron donor among the uncharged kynurenines. IP_GAS_ for XAN is close to that for 3HAA, hence XAN easily abstracts electron, but not H-atom. This possibly makes it a prooxidant with toxic effects [22].

The electron-donating substituents are known to decrease IP and to increase the antioxidant activity [33]. In general, compounds with ionized CO_2_ group have lower IP and higher E_HOMO_ values than their neutral forms (ΔIP_GAS_ = -88.5±11.5 kcal/mol, ΔIP_WATER_ = -8.7±9.5 kcal/mol, without KYNA_OXO_; ΔE_HOMO/GAS_ = 88.6±8.4 kcal/mol; ΔE_HOMO/WATER_ = 7.4±1.6 kcal/mol). KYNA_OXO_ in water solution has lower IP than KYNA_OXO/CO2_. Probably, geometry optimization of charged compounds in the gas phase leads to some distortions in KYNA_OXO/CO2_ structure. In water solution, IP becomes lower for the majority of compounds and higher for the anionic forms. This seems to result from a high dielectric capacity which decreases electrostatic interactions, stabilizes anions, and diminishes electron attraction to cations and neutral molecules. The change of gas–water -E_HOMO_ and -E_LUMO_ is correlated with the change of IP. BDE for XAA_OXO/CO2-_ becomes higher than that for 3HAA and only 2 kcal/mol less than that for XAA_OXO_. The same trend is observed for 3HAA/3HAA_CO2-_ and L-3HOK/L-3HOK_NH3+_. Thus, water solution significantly diminishes the influence of charged groups on BDEs and IPs.

### Kinetic study of kynurenine H-atom donation to *O-group of phenoxyl and methyl peroxy radicals

There are different pathways for ROS inactivation by antioxidants [19]. Most likely, kynurenines quench radicals by donating aromatic hydroxyl H-atom to radical *O-group. We have computationally studied the kinetics of this process for the complexes of four hydroxykynurenines, 3HAA, L-3HOK, XAA_OXO_, and XAA_ENOL_, with phenoxyl radical (Ph-O*) and methyl peroxy radical (Met-OO*). BDE difference for Met-OO* and buthyl peroxy radical is less than 0.7 kcal/mol (levels II, III), hence Met-OO* can be used instead of the radicals with long aliphatic chain to simplify calculations. Ph-O*–DTBP and Ph-O*–DTBA complexes have been also calculated, as well as Met-OO* complex with XAA in ionized form. TSs for reaction pathways were located at level II. Reagent and product complex structures are in good agreement with the results of IRC calculations (RMSD = 0.026±0.015 Å for all complexes and 0.019±0.008 Å for kynurenines' complexes).

The values for the reaction rate and height of activation barrier were calculated in the gas phase and water solution (Table 3). k(T) values are significantly higher than those experimentally shown for phenolic compounds with BDE values of 70–80 kcal/mol, which are about 10^4^−10^7^ M^-1^s^-1^ [19]. This fits the fact that B3LYP underestimates the reaction barrier heights, whereas functional XYG3 is almost as accurate, as the highly precise CCSD(T) method [45].

**Table 3:** Thermodynamic and kynetic parameters of kynurenines H-atom donation to phenoxyl radical and methyl peroxy radical.

	Level II							Level IV									
								Gas phase					Water solution				
	Phenoxyl radical (Ph-O*)																
Compound	ΔE_TS-R_	ΔE_TS-P_	ΔE_P-R_	ΔG_TS-R_	ΔE_TS-R/COR_	ν_i_	k(T)	ΔE_TS-R_	ΔE_TS-P_	ΔE_P-R_	ΔE_TS-R/COR_	k(T)	ΔE_TS-R_	ΔE_TS-P_	ΔE_P-R_	ΔE_TS-R/COR_	k(T)
XAA_ENOL_	8.086	10.273	-2.187	-2.783	5.503	1703.5	5.339x10^10^	9.257	10.948	-1.691	6.674	7.387x10^9^	9.799	11.701	-1.903	7.216	2.962x10^9^
DTBP	7.186	16.069	-8.883	-2.174	5.012	1588.9	1.104x10^11^	8.134	17.294	-9.160	5.960	2.230x10^10^	9.165	17.367	-8.202	6.991	3.910x10^9^
DTBA	6.423	16.208	-9.784	-1.814	4.609	1560.3	2.124x10^11^	7.314	17.209	-9.895	5.499	4.726x10^10^	8.806	17.377	-8.571	6.992	3.805x10^9^
XAA_OXO_	5.808	11.147	-5.338	-2.559	3.249	1613.6	2.212x10^12^	6.968	11.535	-4.567	4.409	3.122x10^11^	8.481	10.705	-2.224	5.922	2.430x10^10^
3HAA	2.660	14.174	-11.514	-2.060	0.600	1295.9	1.444x10^14^	3.463	14.459	-10.996	1.420	3.725x10^13^	5.313	15.111	-9.799	3.252	1.597x10^12^
L-3HOK	2.679	14.162	-11.482	-1.918	0.761	1300.1	1.104x10^14^	3.458	14.511	-11.053	1.540	2.965x10^13^	4.519	14.547	-10.028	2.601	4.950x10^13^
	Methyl peroxy radical (Met-OO*)																
XAA_ENOL_	10.517	13.318	-2.801	-1.270	9.247	1803.5	1.046x10^8^	11.367	14.119	-2.743	10.106	2.454x10^7^	11.948	15.689	-3.741	10.678	9.356x10^6^
XAA_OXO_	8.047	13.822	-5.755	-1.018	7.029	1619.3	3.771x10^9^	8.876	14.648	-5.772	7.858	9.304x10^8^	10.926	15.171	-4.245	9.908	2.926x10^7^
XAA_OXO/CO2-_	0.694	17.795	-17.102	-1.035	-0.341	559.6	2.775x10^14^	1.005	17.326	-16.548	-0.030	2.073x10^14^	7.185	15.080	-7.895	6.150	6.117x10^9^
3HAA	4.922	16.661	-11.739	-0.475	4.447	1309.3	2.211x10^11^	5.148	16.930	-11.782	4.673	1.509x10^11^	6.187	17.798	-11.611	5.711	2.612x10^10^
L-3HOK	4.949	16.450	-11.501	-0.857	4.092	1316.9	4.058x10^11^	5.193	16.847	-11.653	4.336	2.685x10^11^	5.830	17.691	-11.861	4.973	9.171x10^10^

Energies are in kcal/mol, ν_i_ values are in cm^-1^, k(T) values are in M^-1^s^-1^

It is rather difficult to calculate the exact value of the reaction rate, as multiple factors should be considered, and appropriate DFT level should be used [46]. However, the location of TS point calculated for 3HAA–Met-OO* and XAA_OXO_−Met-OO* by B3LYP (level II) is similar to that calculated by XYG3 (level V) (S4 Fig). Thus, even B3LYP with the relatively small basis set II correctly describes the geometry of TS structure. ΔE_TS-R (XYG3)_ for XAA_OXO_ complex is higher than that for 3HAA complex.

For both Ph-O* and Met-OO*, k(T) increases in the following rankings: XAA_ENOL_ < XAA_OXO_ <3HAA ~ L-3HOK (Table 3, Fig 3(C)); the same rankings applies to -ΔE_P-R_.

**Fig 4:**
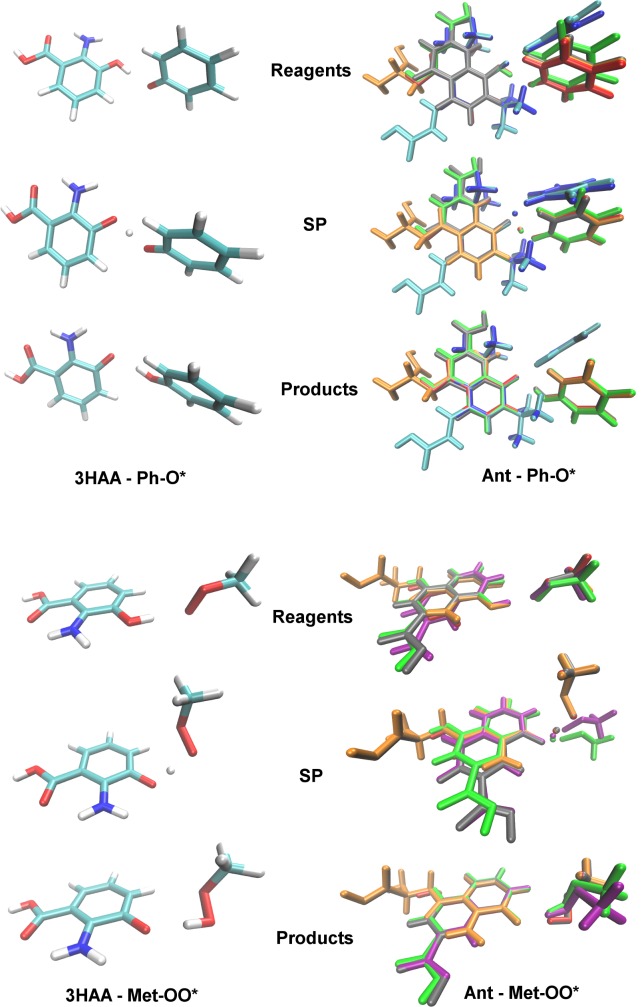
Antioxidants in compex with phenoxyl radical (Ph-O*) and methyl peroxy radical (Met-OO*). Abbreviations: Ant–antioxidant, Ant*–antioxidant radical, SP–saddle point structure. Color scheme, atoms: H–white, C–cyan, O–red, N–blue; color scheme, antioxidants in complex with radicals: 3HAA–red, L-3HOK–orange, XAA_OXO_−grey, XAA_ENOL_−green, XAA_OXO/CO2-_ –purple, DTBP–blue, DTBA–cyan.

The structures of radical complexes with 3HAA, L-3HOK, and XAA_OXO_ are very similar (Fig 4, Table 4). For kynurenines in complex with Ph-O*, aromatic rings of reagents and products form the plane angle of ~50–70°. The geometry of Ph-O*–DTBP is very different: aromatic rings are nearly perpendicular in reagent and product complexes. Ph-O*–DTBP and Ph-O*–DTBA complexes are rather similar. For 3HAA, L-3HOK, and XAA_OXO_ in complex with Met-OO*, the radical rotates in space along with the attachment of H-atom, so O-O* and C-O bonds in Met-OOH become nearly perpendicular to those in reagent complexes. The direction of Met-OO* rotation is different in complexes with XAA_ENOL_ and XAA_OXO/CO2._ In all cases, O…H…O bond significantly shortens upon the TS formation.

**Table 4:** Geometry of antioxidant in complex with radicals.

	Ph-O*			Met-OO*		
	O. . .H. . .O length (Å)	O. . .H. . .O angle (°)	Plane angle^a^ (°)	O. . .H. . .O length (Å)	O. . .H. . .O angle (°)	Torsion^b^ (°)
Reagents (Ant-OH. . .*O-Rad)						
3HAA/L-3HOK/XAA_OXO_	2.75±0.01	168.8±1.1	67.2±4.6	2.89±0.03	176.8±1.3	101.0±7.1
XAA_ENOL_	2.77	164.9	50.8	2.90	174.4	105.4
XAA_OXO/CO2-_				2.70	175.6	114.3
DTBP	2.81	160.1	86.4			
DTBA	2.79	162.6	64.0			
SP (Ant-O. . .H. . .O-Rad)						
3HAA/L-3HOK/XAA_OXO_	2.41±0.03	177.8±1.6	58.3±1.7	2.38±0.04	177.8±1.6	350.7±8.8
XAA_ENOL_	2.40	179.0	48.6	2.39	171.1	134.1
XAA_OXO/CO2-_				2.48	174.9	120.9
DTBP	2.39	168.4	63.9			
DTBA	2.39	168.3	69.6			
Products (Ant-O*. . .HO-Rad)						
3HAA/L-3HOK/XAA_OXO_	2.79±0.04	166.7±3.7	64.4±4.6	2.82±0.04	163.9± 0.1	27.8±3.2
XAA_ENOL_	2.80	165.4	62.0	2.81	166.2	86.6
XAA_OXO/CO2-_				2.70	175.6	114.3
DTBP	2.81	171.5	82.9			
DTBA	2.81	170.0	89.7			

Abbreviations: Ant–antioxidant, Rad–radical. a. The plane angle was calculated as the angle between planes formed by C(O*)_1_, C_3_, and C_5_ atoms of the antioxidant and radical aromatic rings. b. Torsion was calculated as torsion between antioxidant C-O* and radical *O-C bonds.

The influence of solvent and partial charges' distribution on antioxidant activity depends on whether HAT or PCET is the dominant mechanism of H transfer. The increase in H-atom charge in the TS compared to the parent antioxidant is specific for PCET [20]. The interaction of phenolic antioxidants with tert-buthyl-peroxy radical is known to occur via PCET [47]. PCET-TS is stabilized by the enhanced spin density (SD) and electron density on radical O_2_ and O_3_ atoms. Thereby (O_3_+O_2_-O_1_) negative charge and Δ(O_3_+O_2_-O_1_)_TS-R_ negative charge correlate with the reaction rate [47]. In our study, positive charge on H-atom increases in all TSs (ΔQ(H) > 0), and the negative charge on O atoms in the gas phase moves towards the free radical (Δ(dQ)_TS-R_ < 0) (Table 5). In the gas phase, there is a strong correlation between ΔE_TS-R_ and SD on O_1_ and C_PARA_ atoms of antioxidants. Hence the high SD on these atoms decreases the reaction rate.

For Ph-O* complexes in water solution, the decrease of the negative charge on radical O atoms (Δ(dQ)_TS-R_) correlates with the growth of ΔE_TS-R_, as it is typical for PCET. There is a negative correlation between ΔE_TS-R_ and E_TS-SOMO_. Thus, E_SOMO_ may serve to predict the reaction rate, as shown by Nikolic [47].

**Fig 5:**
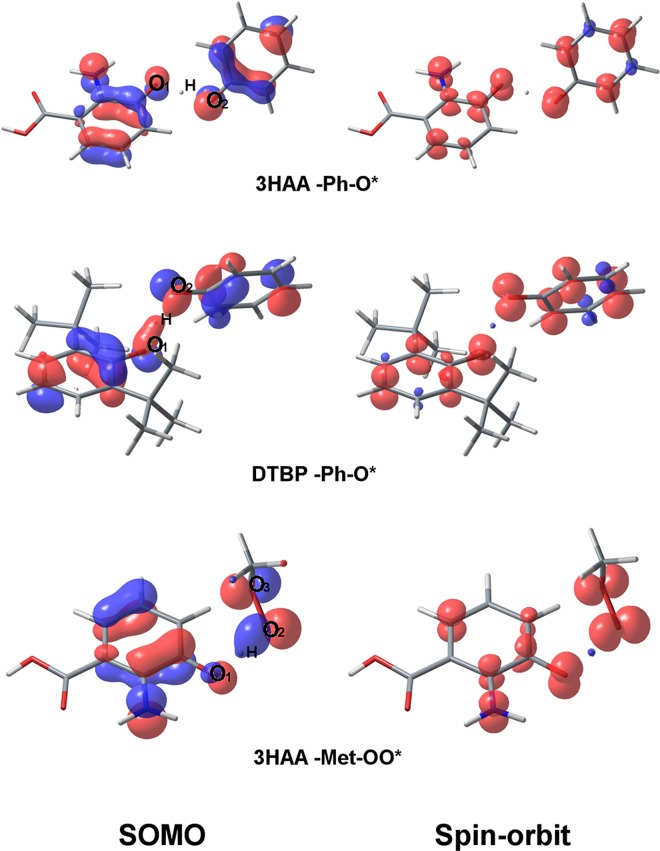
SOMOs and spin-orbits of kynyrenine-radical TSs. Color scheme, atoms: H–white, C–cyan, O–red, N–blue. Isosurface values: 0.05 –for SOMOs, 0.005 –for spin-orbits.

The geometry of TS SOMO and spin-orbit on O_1_ and O_2_ differs from both classical σ- and π-orbitals: p-orbitals on O atoms form a sharp angle projected to plane passing through H atom perpendicular to O…H…O bond (Fig 5). In Ph-O*–kynurenines' complexes, p-orbitals are nearly parallel to this plane and perpendicular to O…H…O bond. In Ph-O*–DTBP complex, O_1_ and O_2_ protrude parts of the electron clouds towards H, and in Met-OO*–kynurenines' complexes, the angle between O…H…O and O_1_ p-orbital is close to 45°. Hence kynurenine's SOMO in Ph-O* complexes is closer to π-orbital than in Met-OO* complexes.

**Table 5:** E_SOMO_ (kcal/mol), charges, and spin densities (e) for the transition structures of kynurenines in complex with free radicals.

	Gas phase							Water solution						
Complexes	E_SOMO_	Q(H)	ΔQ(H)	dQ(O)	Δ(dQ)_TS-R_	SD(O_1_)	SD(C_PARA_)	E_SOMO_	Q(H)	ΔQ(H)	dQ(O)	Δ(dQ)_TS-R_	SD(O_1_)	SD(C_PARA_)
Phenoxyl radical (Ph-O*)														
XAA_ENOL_	-125.941	0.663	0.208	-0.007	-0.123	0.139	0.200	-127.823	0.669	0.204	0.098	-0.005	0.069	0.113
DTBP	-132.091	0.564	0.246	0.065	-0.042	0.146	0.166	-133.346	0.582	0.250	0.027	-0.013	0.133	0.156
DTBA	-133.848	0.558	0.227	0.079	-0.033	0.112	0.139	-132.655	0.576	0.225	0.071	0.027	0.080	0.092
XAA_OXO_	-131.463	0.672	0.170	0.077	-0.104	0.109	0.138	-130.271	0.666	0.162	0.149	0.031	0.033	0.045
3HAA	-120.482	0.663	0.184	0.059	-0.106	0.107	0.104	-121.297	0.658	0.179	0.003	-0.122	0.111	0.111
L-3HOK	-120.419	0.662	0.177	0.060	-0.110	0.106	0.099	-120.795	0.657	0.174	-0.011	-0.143	0.116	0.113
Methyl peroxy radical (Met-OO*)														
XAA_ENOL_	-133.659	0.554	0.107	0.043	-0.314	0.178	0.232	-135.793	0.562	0.096	0.050	-0.337	0.165	0.248
XAA_OXO_	-142.696	0.532	0.039	0.148	-0.286	0.161	0.167	-140.186	0.535	0.029	0.150	0.289	0.148	0.169
XAA_OXO/CO2-_	-65.951	0.562	0.058	0.030	-0.151	0.098	0.115	-131.275	0.555	0.057	0.222	-0.205	0.069	0.093
3HAA	-130.898	0.519	0.048	0.121	-0.306	0.146	0.127	-130.898	0.520	0.038	0.030	-0.392	0.146	0.140
L-3HOK	-130.898	0.516	0.046	0.124	-0.305	0.145	0.125	-130.710	0.516	0.033	0.027	-0.401	0.146	0.141
Pearson correlation with ΔE_TS-R_														
Ant–Ph-O*	**-0.729**	**-0.387**	**0.610**	**-0.393**	**0.295**	**0.762**	0.951	-0.879	**-0.361**	**0.572**	**0.704**	0.918	**-0.419**	**-0.024**
Ant–Met-OO*	**-0.790**	**-0.046**	**0.516**	**0.185**	**-0.748**	0.965	0.911	**-0.865**	**0.662**	**0.552**	**0.147**	**0.519**	**0.392**	**0.789**
All	-0.717	**-0.124**	**0.204**	**-0.006**	**-0.185**	0.765	0.905	-0.779	**-0.147**	**0.044**	**0.399**	**0.419**	**0.085**	**0.484**

O_1_ is the atom in antioxidant from which H is abstracted, O_2_ is the atom in free radical to which H is transferred, C_PARA_ is the atom in para-position of the antioxidant aromatic ring relative to C(O_1_). Q is Mulliken partial charge; dQ(O) is Q(O_2_)+Q(O_3_)-Q(O_1_) for Met-OO* and Q(O_2_)-Q(O_1_) for Ph-O*; Δ(dQ)_TS-R_ is the difference between dQ(O) of TS and reagents; SD is spin density. Pearson correlation was calculated for the corresponding value and ΔE_TS-R_ (level IV) of antioxidants (Ant) in complex with Met-OO* (n = 5), Ph-O* (n = 6), and both free radicals (n = 11). Bold: the values which are not statistically significant (p>0.05).

Partial charges Q and ΔQ on H are higher, and the negative charge displacement to O_1_ is lower for Ph-O* complexes than for Met-OO* complexes. Thus, PCET seems to be more preferable for kynurenines' reaction with Ph-O* than for their reaction with Met-OO*. HAT may also occur in both cases, however, SOMO geometry and charges distribution character indicate that it is not the chief mechanism of H transfer for the studied complexes.

### Thermodynamic study of methyl peroxy radical addition to the aromatic ring of kynurenine radicals

**Fig 6:**
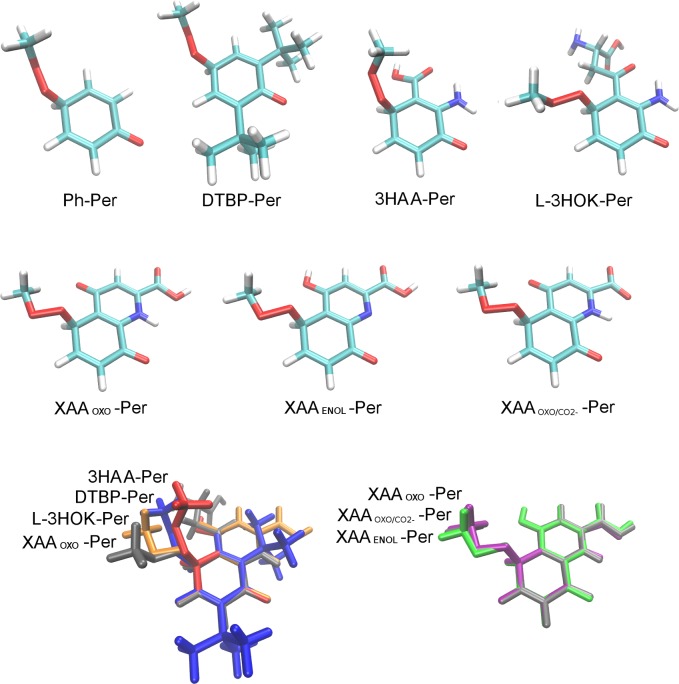
Products of Met-OO* addition to the aromatic ring of antioxidants. Color scheme, atoms: H–white, C–cyan, O–red, N–blue; color scheme, the products of Met-OO* addition to antioxidant radicals: 3HAA–red, L-3HOK–orange, XAA_OXO_−grey, XAA_ENOL_−green, XAA_OXO/CO2-_ –purple, DTBP–blue.

Another possible way of free radical quenching is its addition to the aromatic ring of the antioxidant radical. We have modeled the products of Met-OO* addition to the aromatic ring of phenoxyl and kynurenines radicals at para-position relative to O* atom (Fig 6, Table 6). The orientation of side chain Met-OO group varies, being closer for the different forms of XAA than for the different antioxidants.

**Table 6:** Thermodynamic parameters (kcal/mol) of methyl peroxy radical addition to the aromatic ring of kynurenines radicals.

	Level II		Level IV	
			Gas phase	Water solution
Radical	ΔE_P-R_	ΔE_P-R/COR_	ΔE_P-R/COR_	ΔE_P-R/COR_
Phenoxyl*	-49.289	-33.125	-33.237	-29.494
XAA_ENOL_*	-30.129	-14.485	-12.340	-5.364
DTBP*	-28.240	-12.377	-10.978	-4.887
XAA_OXO_*	-27.560	-11.541	-9.313	-1.875
XAA_OXO/CO2-_*	-26.759	-10.703	-8.589	-1.307
L-3HOK*	-25.127	-8.180	-6.047	1.492
3HAA*	-20.264	-5.652	-3.551	3.405

In the gas phase, all reactions are thermodynamically favorable (ΔE_P-R/COR_ < 0), in contrast to H abstraction. In water solution, radical addition to 3HAA and L-3HOK radicals is slightly unfavorable. The rankings of -ΔE_P-R_ and -ΔE_P-R/COR_ are the same at all levels: 3HAA* < L-3HOK* < XAA_OXO/CO2-_* < XAA_OXO_* < DTBP* < XAA_ENOL_* < Ph-O*. It is reverse to the rankings of -ΔE_P-R_ and k(T) for H-atom donation: the affinity to Met-OO* is minimal for 3HAA* and maximal for phenoxyl radical. XAA_OXO/CO2-_* is less active than XAA_OXO_* and more active than L-3HOK*, both in the gas phase and in water solution. Thus, high Met-OO* scavenging activity of L-3HOK and 3-HAA is unlikely to be explained by Met-OO* addition to the aromatic rings of kynurenine radicals.

### Lipophilicity estimation for kynurenines and phenolic antioxidants

The antioxidant power of a substance depends not only on its chemical properties, but also on its ability to penetrate into the surroundings where it displays its antioxidant activity. To inhibit lipid peroxidation, a substance should have high lipophilicity. It can be measured as a logP value, where P is the octanol-water partition coefficient [48]. We used the Molinspiration method of logP calculation reported to be robust and precious.

Among the antioxidants studied, substituted phenols, such as DTBP and DTBA, have maximal lipophilicity, whereas the kynurenines' ions have higher water solubility compared to ASC (Table 7). Lipophilicity decreases in the following rankings: AA > 3HAA> XAA_OXO_ > QUIN > L-3HOK; the rankings are the same for kynurenines' carboxylic anions. Hence XAA should penetrate through lipid bilayer better than 3HOK and 3HAA. This fact does not fit with the lower rate of XAA reaction with peroxy radicals, which is rather explained by the higher rate of H donation.

**Table 7:** Antioxidants lipophilicity.

Compound	LogP	TPSA (Å^2^)	Volume (Å^3^)
DTBP	5.04	20.23	224.44
DTBA	4.96	57.53	278.85
DIBP	4.22	20.23	225.56
DIBA	4.15	57.53	279.98
KYNA_ENOL_	1.98	70.42	159.90
XAA_ENOL_	1.72	90.65	166.92
Phenol	1.46	20.23	92.06
AA	1.46	63.32	122.33
3HAA	1.20	83.55	130.35
2-aminophenol	1.15	46.25	103.35
KYNA_OXO_	0.68	70.16	159.01
XAA_OXO_	0.42	90.39	167.02
Methane	0.34	0.00	28.64
QUIN	0.25	87.49	133.89
Water	-0.29	29.27	19.33
DXAN	-0.98	119.71	339.88
ASC	-1.40	107.22	139.71
XAN	-1.47	193.66	334.29
AA_CO2-_	-2.13	66.55	119.59
L-KYN	-2.18	106.42	186.24
KYNA_OXO/CO2-_	-2.39	72.99	156.26
3HAA_CO2-_	-2.39	86.38	127.61
L-3HOK	-2.45	126.64	194.25
XAA_OXO/CO2-_	-2.65	93.22	164.28
L-KYN_ZI_	-2.78	110.86	184.29
QUIN_CO2-_	-2.82	90.32	131.15
L-3HOK_ZI_	-3.05	131.09	192.31
L-3HOK_NH3+_	-4.78	128.26	195.05

ZI–zwitterionic form (αNH_3_^+^, αCO_2_^-^)

Topological polar surface area (TPSA) is a molecular descriptor numerically close to PSA which can be used for the prediction of passive transport through membranes in intestines and blood-brain barrier [49]. Drugs that penetrate the brain by passive absorption typically have PSA < 70 Å^2^, while the most non-CNS active drugs have much larger PSA values up to 120 Å^2^ [50]. In our study, TPSA is less than 120 Å^2^ for all compounds except L-3HOK, so they are more or less capable of penetrating passively through the plasma membrane. TPSA is minimal for phenolic antioxidants, which should be easily absorbed in intestines and penetrate into the brain. It is significantly higher for 3HOK (> 120 Å^2^) than for 3HAA (< 90 Å^2^), while XAA has the intermediate TPSA. Hence in the case of absence of specific carriers 3HAA should more actively penetrate through lipid bilayer than 3HOK.

## Discussion

A great need in studies of biochemical properties of KP is promoted by the fact that this very pathway plays an overwhelming role in physiology and pathology. Dysregulation of this pathway, resulting in hyper- or hypofunction of active metabolites, is associated with neurodegeneration and other disorders, such as depression and schizophrenia [51], diabetes mellitus [52,53], attention-deficit hyperactivity disorder [54], and cataract [13]. Some KP metabolites are neuroactive, while others are molecules with prooxidant and antioxidant properties [3]. Therefore, it is necessary to understand the molecular and biophysical mechanisms of kynurenines' activity to elaborate the strategy of disorders' prevention and therapy.

In this study, we investigated the antioxidant activity of kynurenines, namely their ability to donate electron and H atom. The hydroxyl group BDE and adiabatic IP are the most important determinants for the radical scavenging activity of substituted phenols [55]. According to our data, the antioxidant properties of 3HOK and 3HAA are determined by their 2-aminophenolic moiety. For the uncharged hydroxykynurenines, BDE and IP are maximal for KYNA_ENOL_ and miminal for DXAN, both in the gas phase and in water solution. 3HOK and 3HAA have lower BDE and IP than XAA, ascorbic acid, and some phenolic antioxidants, such as DTBP and DTBA. Aromatic OH group diminishes IP values for 3HOK, 3HAA, and XAA relative to KYN, AA, and KYNA. Our results confirm the correlation between BDE and IP also shown by Borges [56]. Negatively charged carboxylic group significantly diminishes BDE and IP values, while positively charged amino group enhances them. This phenomenon can be explained by electron-donating and withdrawing effects of substituents [18]. The effects of charged groups are significantly more pronounced in the gas phase than in water solution. Basis set and the type of density functional had a little effect on the rankings for BDE values of kynurenines.

Adiabatic IP strongly correlates with -E_HOMO_, confirming that Koopmans' theorem can be used to calculate IP at DFT level [44]. However, B3LYP significantly underestimates the absolute values of E_HOMO_. We have used the tuned LC-BLYP range-separated functional to compute E_HOMO_, E_LUMO_, H-L gap, and IP for several antioxidants, including three hydroxykynurenines. The tuned LC-BLYP gives significantly higher absolute values for E_HOMO_ and H-L gap, also, there is a minor difference between -E_HOMO_ and adiabatic IP. However, the rankings for kynurenines' E_HOMO_ values are the same as for those calculated using B3LYP. Also, BDEs calculated with B3LYP and HCTH/407 are highly correlated. B3LYP is significantly faster than the high quality functionals partly based on perturbation theory, such as XYG3. B3LYP contains less empirical parameters than HCTH/407, thereby it seems to be more universal. B3LYP has been successfully used to model both thermodynamic and kinetic properties of free radicals [18,32,33]. Thus, we used B3LYP in the majority of our calculations. At the same time, using LC-BLYP and other range-separated functionals may be favorable to predict the exact values for frontier orbital energies.

High radical stability and even spin distribution are among the factors predisposing low BDE and IP values [55]. Conjugated bonds system facilitates electron delocalization after HAT or single electron transfer (SET). Standard deviation of SD in kynurenine radicals is correlated with BDE; however, there is no significant correlation between SD and IP. BDE for kynurenines is correlated with SD on O* and C_PARA_ atoms. SD on antioxidant O* atom in TS complex with Met-OO* is also strongly correlated with the height of the activation barrier. The same is true for O_PARA_ atom in phenolic antioxidants [47].

The rate of H-atom donation to phenoxyl and methyl peroxy radicals is correlated with BDE: 3HAA and 3HOK are more active radical scavengers than XAA. Likewise, for phenolic compounds donating H to hydroxyl radical, the rate constant is negatively correlated with O-H bond straight, IP, and SET enthalpy [57]. The rankings for free energies of radical addition to kynurenine radicals in para-position relative to OH group is reverse: 3HAA* radical has the lowest affinity to Met-OO*. Thus, high antioxidant activity of 3HAA and 3HOK relative to XAA [15] rather can be explained by their lower BDEs and higher rates of H-atom donation to peroxy radical. PCET seems to be the chief mechanism for H donation by kynurenines to phenoxyl radical and, probably, to Met-OO* radical. Studies of oxidations of O-H bond usually invoke stepwise oxidation, where the positive and negative charges are transferred separately. Here, there may be a complex dependence of BDE and k(T) on solvent, biochemical surroundings, and pH [58].

Low BDE and IP values are not sufficient for Ant-OH to be a powerful antioxidant without toxic side effects. Some of the necessary conditions include: 1) O_2_ should not abstract H from Ant-OH; 2) Ant-OH should react with ROO* much faster than ROO* with R-H; 3) Ant-O* should not abstract H from R-H at an appreciable rate; 4) Ant* should not react with O_2_ to produce AOO*; 5) Ant-OH and its products should not be toxic [21]. Though we did not concentrate on the study of these properties, our calculations performed for 3HAA at level II have shown that:

1. for H abstraction from 3HAA by O_2_ (in triplet form): ΔE_P-R_ is 11.0 kcal/mol, ΔE_TS-R/COR_ is 16.4 kcal/mol, k(T) is 3.1 х 10^2^ M^-1^s^-1^;
2. for H abstraction from aliphatic ethane by Met-OO*: ΔE_P-R_ is 22.1 kcal/mol, ΔE_TS-R/COR_ is 25.8 kcal/mol, k(T) is 6.1 х 10^−5^ M^-1^s^-1^;
3. for H abstraction from ethane by 3HAA*: ΔE_P-R_ is 32.3 kcal/mol, ΔE_TS-R/COR_ is 34.0 kcal/mol, k(T) is 5.3 х 10^−11^ M^-1^s^-1^.

Hence O_2_ can abstract H from 3HAA, but the reaction is dramatically slower than H abstraction by Met-OO* (2.2 x 10^11^ M^-1^s^-1^; Table 3). The aliphatic ethane interacts with Met-OO* faster than with 3HAA, and 3HAA interacts with Met-OO* dramatically faster than with ethane. Only the reaction of 3HAA with Met-OO* is thermodynamically favorable, so O_2_ or free radicals must be in high concentration to hinder the protective action of 3HAA. Therefore, it seems to be a potent antioxidant, as well as 3HOK.

At the same time, the ability of 3HAA and 3HOK to form dimers leads to the production of toxic free radicals which damage the cell [23,26,22]. 3HAA undergoes three successive one-electron oxidative reactions: 1. conversion to semiquinoneimine (hydroxyl H abstraction); 2. conversion to quinoneimine (amine H abstraction); 3. two quinoneimine molecules condensation to cinnabarinic acid [59,60]. The rate of 3HAA oxidation increases exponentially with increasing pH [59]. This corresponds to our data that BDE decreases and k(T) for H-atom donation increases for compounds with ionized carboxylic group. 3HOK autoxidation is similar to that of 3HAA [29]. BDE for 3HOK N-H is significantly higher than for 3HOK O-H, being 99.5 and 77.2 kcal/mol, respectively (level III). Both of them are smaller for semiquinoneimine, becoming 91.8 and 69.4 kcal/mol after the other H-atom abstraction. Hence the first stage of oxidation facilitates the second one. o-Quinoneimine which is synthesized at the second stage may be responsible for the prooxidant effects of 3HOK and 3HAA [29]. The enzymatic oxidation of o-aminophenols leads to the concomitant reduction of oxygen to water [25]. Non-enzymatic oxidation produces the toxic reactive forms of oxygen. Therefore, it may be therapeutically important to enhance the antioxidant power of hydroxykynurenines by inhibiting their non-enzymatic dimerization and/or stimulating the enzymatic dimerization.

The inhibition of tryptophan 2,3-dioxygenase, the key enzyme of KP, is neuroprotective in Drosophila *huntingtin* (*htt*) mutant. Feeding flies by 3HOK alone, in the absence of mutant HTT, did not cause neurodegeneration [14]. Thus, the high level of 3HOK is toxic, yet, it may be not sufficient for neurodegeneration which also requires the additional factors, such as the lack of neuroprotectant KYNA. Both 3HOK and 3HAA inhibit the spontaneous lipid peroxidation in the brain [61]. The dual redox activity of 3HOK makes it prooxidant at low concentrations (5–20 μM) and antioxidant at higher concentrations (100 μM) in the rat striatum slices. 3HOK seems to be a redox modulatory molecule which stimulate the increase in glutathione reductase and glutathione S-transferase activities [62]. Interferon-γ induces TRP degradation along the KYN pathway in mononuclear blood cells and inhibits the oxidation of low density lipoprotein (LDL). 3HAA inhibits LDL oxidation in submicromolar concentrations, probably being a catalyst for the other antioxidants [63]. It is a highly efficient coantioxidant for plasma lipid peroxidation which can be initiated by α-tocopherol radical (α-TO*).

3HAA in low concentration (5μM) inhibits α-TO* production and accumulation of lipid peroxides. 3HOK inhibitory efficacy is the same as for 3HAA, but AA lacking the phenolic group can not reduce α-TO* [16]. This fits with low BDE for kynurenines having phenolic group. Monocytes in human blood can release 3HAA in concentration up to 30 μM [63]. Thus, 3HOK and 3HAA display the antioxidant activity under physiological conditions, not only in the brain, but also in blood plasma regulating the process of atherogenesis. In contrast to 3HAA, the antioxidant properties of XAA are in relation to its ability to chelate the transition metals which induce LDL oxidation [64]. Thus, kynurenines' action on redox conditions and physiological processes depends on their level in organism. The lack of kynurenines in Drosophila mutant *vermilion*, as well as the excess of 3HOK in *cardinal*, leads to the progressive loss of 3 h memory performance under conditioned courtship suppression paradigm [65,12]. The lack of kynurenines redox activity might partially cause these effects.

Not only BDE and IP define the antioxidant power of substances, but also their ability to pass through the biological barriers, mainly the lipid bilayers. The lipophilicity of kynurenines is low, compared to phenolic antioxidants, due to their polar and charged groups. Hence they should rather act in water environment than in membrane. Besides, their surfaces are quite large, that should hamper their penetration through intestine and blood-brain barriers. Indeed, 3HAA, KYNA, and QUIN poorly cross the blood-brain barrier by passive diffusion, but KYN and 3HOK are taken up into the brain by a large neutral amino acid carrier [66]. AA easily penetrates into the brain by passive diffusion that can be explained by its high logP and low TPSA values. Kynurenine pathway enzymes in the brain are preferentially localized in astrocytes and microglia; however, the cerebral pathway is driven mainly by blood-borne KYN [2]. Thus, 3HOK, KYN, and AA may play an important role in the brain both as prooxidants and antioxidants.

In our study, we did not consider several important factors affecting the antioxidant power of kynurenines, such as: 1) thermodynamics and kinetics of 3HOK and 3HAA dimerization; 2) energy and rate of proton abstraction from antioxidant OH group; 3) interaction between solvent and OH group of kynurenines; 4) hydrogen bond formation between functional kynurenine groups; 5) steric effects of side-chain groups on free energy and rate of kynurenine interaction with radicals, etc. Other functional groups of kynurenines can also donate H-atom, such as the 3HOK aromatic NH_2_ group, which BDE was shown to be significantly higher than that for OH group. It would be interesting to evaluate the activity of kynurenines and phenolic antioxidants in their native surroundings, such as lipid bilayer, affecting the dielectric capacity and hydrophobic interactions. Consideration of these factors is a task for the future.

## Methods

### Preparation of initial structures for DFT calculations

The structures of 2-aminophenol, anthranilic acid (AA), ascorbic acid (ASC), kynurenic acid (KYNA), L-kynurenine (L-KYN), D-3-hydroxykynurenine (D-3HOK), L-3-hydroxykynurenine (L-3HOK), quinolinic acid (QUIN), 2,6-di-tert-butylphenyl-4-hydroxymetylphenol, xanthommatin (XAN), dihydroxanthommatin (DXAN), and xanthurenic acid (XAA) were taken from PubChem Compound database [67]. The structures of phenol, 2,6-di-isobutylphenol (DIBP), 2,6-di-tert-butylphenol (DTBP), β-(4-hydroxy-3,5-di-isobutylphenyl) propenoic acid (DIBA), β-(4-hydroxy-3,5-di-tert-butylphenyl) propenoic acid (DTBA), and 3-hydroxyanthranilic acid (3HAA) were constructed on the base of PubChem structures using Vega ZZ 3.0.3 [68]. The systematic conformational search of low-energy geometry for the constructed structures was performed using Avogadro [69].

The ionic forms for kynurenines with α-carboxylic group (total charge -1) were modeled as well as the uncharged forms. The major forms for 3HAA and XAA at physiological pH (7.4) are the forms with the ionized carboxylic group, while KYN and 3HOK are mainly in zwitterionic form with ionized α-amino and α-carboxylic group [70].

### BDE and IP calculations

All quantum chemical calculations were performed using Firefly 8.1.0 partially based on the GAMESS (US) [71] source code. Firefly 8.1.0 was kindly provided by Alex A. Granovsky [72]. The geometries of molecular structures with neutral total charges were fully optimized using density functional theory (DFT) at 6-31G(d) level (I), B3LYP/6-31G(d) level (II), and B3LYP/6-311G(d,p) level (III) [73–75]. B3LYP1 version of B3LYP was used. Highly parameterized functional HCTH/407 [76] was also used to calculate BDE values for compounds at level II. Closed shell configurations were calculated with restricted Hartree-Fock or DFT methods; open shell configurations were calculated with unrestricted Hartree-Fock or DFT methods. All closed shell molecules were calculated in a singlet state, whereas doublet state was used for free radicals. The symmetry point group was set as C1 for all compounds. Hessian matrix, vibrational frequencies, and thermal corrections to the enthalpy were calculated with the same methods. The enthalpies and free energies were obtained from the vibrational frequency calculations at 298.15 K, using unscaled frequencies. In order to calculate the adiabatic ionization potential (IP), cation-radical forms of corresponding molecules were fully optimized at level III. BSSE correction [77] was performed for several compounds at level III. The nature of all stationary points was determined by evaluating the vibrational frequencies. Standard deviation of Mulliken spin density (δSD) was used as an estimator of electron delocalization on the radicals.

The following energy parameters were estimated:

1. The energies of the highest occupied molecular orbital (E_HOMO_) related to electron donating capacity or the ionization potential (IP) and the lowest unoccupied molecular orbital (E_LUMO_) related to the electron accepting capacity or the electron affinity.
2. H-L gap: the difference between E_HOMO_ and E_LUMO_ related to the chemical hardness and the ability of compound to participate in oxidation-reducing reactions.
3. Homolytic bond dissociation enthalpy (BDE) and corrected enthalpy (BDE_COR_) for O-H bond:
  $$BDE=E_{RAD}+E_{H}–E_{W}$$ (2)
  where E_W_ is the total energy of the whole molecule, E_RAD_ is the total energy of the radical after H-atom abstraction, E_H_ is the total energy of H radical;
  $$BDE_{COR}=BDE+H_{T}$$ (3)
  $$H_{T}=H_{RAD}+H_{H}–H_{W}$$ (4)
  where H_T_ is the thermal correction to enthalpy, H_RAD_, H_H_, H_W_ are the thermal contributions to enthalpies for the antioxidant radical, H radical, and the whole molecule, respectively.
4. Ionization potential:

$$IP=E_{CAT}–E_{W}$$ (5)

where E_CAT_ is the energy of cation radical after single electron abstraction (or the neutral form for compounds with ionized carboxylic group), E_W_ is the total energy of the whole molecule.

BDEs for methane, water, phenol, 2-aminophenol, water-soluble antioxidant ASC (uncharged form), and phenolic antioxidant DTBP were used as standards and reference points to estimate the relative activities of antioxidants. For symmetric phenol and DTBP radicals, there were significant deviations of BDE from the experimental values. To exclude the possible artefacts, BDE was also calculated for several structural analogues of DTBP–DTBA, DIBP, and DIBA, which are believed to have similar BDE values.

Zwitterionic form is not stable in the gas phase, therefore, the optimization of KYN and 3HOK in the neutral form was performed. To check the influence of positively charged group on BDEs and IPs, calculations were performed for L-3HOK with aromatic NH_3_^+^ group (total charge +1). 3HAA and AA cations with ionized carboxylic group are not stable in the gas phase, therefore, their optimization was performed in water solution at level IV (see below), without cavitation, dispersion and repulsion free energies.

NWChem software [78] was used to calculate E_HOMO_, E_LUMO_, H-L gap, and IP with the help of the tuned range-separated hybrid functional LC-BLYP for five compounds optimized with B3LYP (III). The tuning of optimal range-separation parameter μ was done as in [40]: the single point energies were calculated using basis set III for antioxidant's cation, anion, and neutral form for different values of μ ranging from 0.05 to 0.9 with increments of 0.05, and then the optimal parameter was obtained by minimizing the following function:

$$J^{2}(\mu )={\left[E^{\mu }{}_{HOMO}\left(N\right)+IP^{\mu }\left(N\right)]{}^{2}+[E^{\mu }{}_{HOMO}\left(N+1\right)+IP^{\mu }\left(N+1\right)\right]}^{2}$$ (6)

where N is the number of electrons in antioxidant.

For XAA_OXO_, J^2^(0.1) and J^2^ (0.15) were obtained by spline interpolation due to the problem with DFT convergence. The curves for *J*^*2*^(μ*)* are shown in S5 Fig; the minimum of each curve (optimal μ, see Table 1) was obtained by spline interpolation.

Firefly 8.1.0 and Gaussian 98 [79] give almost equal values of total energy for phenol and significantly different values for phenoxyl radical. Gaussian 98 uses Harris functional for the initial orbital guess by default instead of extended Huckel calculations used by Firefly. Harris functional is a nonself-consistent approximation to Kohn-Sham density functional theory [80], hence electron correlations should be partially taken into account. Nevertheless, the differences between Gaussian 98 (S1 Table) and Firefly (Table 1) BDE values are generally small (0.092±0.03 kcal/mol; p < 0.05, n = 16, without phenol).

### Transition structures optimization and reaction rate calculations

For antioxidants in complex with phenoxyl radical (Ph-O*) and methyl peroxy radical (Met-OO*), transition structures (TSs) and corresponding local minima were optimized at level II. Intrinsic reaction coordinates (IRC) calculations [81] were performed for all TS species at the same level to confirm that anticipated reagent (R) and product (P) are connected to TS on potential energy surface. The products of the Met-OO* addition to antioxidant radical in para-position were optimized at level II. ΔE_COR_ is corrected reaction activation energy:

$$\Delta E_{TS-R/COR}=\Delta E_{TS-R}+\Delta G_{TS-R}$$ (7)

$$\Delta E_{TS-R}=E_{TS}–E_{R}$$ (8)

$$\Delta G_{TS-R}{=}_{}G_{TS}–G_{R}$$ (9)

$$\Delta E_{P-R/COR}=\Delta E_{P-R}+\Delta G_{P-R}$$ (10)

$$\Delta E_{P-R}=E_{P}{–}_{}E_{R}$$ (11)

$$\Delta G_{P-R}{=}_{}G_{P}–G_{R}$$ (12)

where ΔG_TS-R_ and ΔG_P-R_ signify thermal correction to free energy at 298.15 K; E_TS_, E_R,_ and E_P_ are total energies of TS, R, and P; G_TS_, G_R_, and G_P_ are thermal contributions to free energies of TS, R, and P.

The rate of reaction (M^-1^s^-1^) between antioxidant and radical was calculated as in [32,46] using conventional TS theory:

$$k\left(T\right)=I\;x\;\left(k_{B}T/h\right)\;x\;[exp(-\Delta E_{TS-R/}{}_{COR}/RT)]\;x\;24.3\;x\;A\left(T\right)$$ (13)

where I is the reaction pathway degeneracy (equal to 1 for the all compounds), k_B_ is Boltzmann's constant, h is Planck's constant, 24.3 is a multiplier used to convert the units from 1 atmosphere standard state to 1 M standard state, and A(T) is a temperature-dependent factor which corresponds to quantum mechanical tunneling, approximated by the Wigner method [82]:

$$A\left(T\right)=1+\left(1/24\right)\;x\;{\left(1.44\nu_{i}/T\right)}^{2}$$ (14)

where ν_i_ is the imaginary frequency (cm^-1^) whose vibrational motion determines the direction of the reaction.

Atom coordinates of the optimized structures are given in S1 Dataset.

### Single point energy calculations in the gas phase and water solution

For the structures optimized at level II (TSs) and III (all other structures), the single point energy calculations were performed at B3LYP/6-311+(O)+G(d) level with diffuse sp functions added only to O atoms (level IV) or at B3LYP/6-311++G(d,p) level (V), both in the gas phase and in water solution at 298.15 K using dielectric polarizable continuum model (DPCM) [83]. Due to the DFT convergence problem, the calculations were performed at level V only for five compounds (L-KYN, L-3HOK, 3HAA, KYNA, and AA). Pearson correlation coefficient R for IPs calculated at levels IV and V is 0.999. Hence the lack of H(p) polarization functions and C, N(sp) diffuse functions at level IV did not change the rankings of IP values for different kynurenines. Single point energy calculations were performed for 3HAA–Met-OO* and XAA_OXO_−Met-OO* IRC at level V in the gas phase using XYG3 functional [45].

The values of the total free energy in solvent were used to calculate ΔE for the compounds in water solution. Since the value of the thermal correction to BDE (III) was very similar for different compounds (-6.645±0.260 kcal/mol, p < 0.05, n = 16), it was not considered. Also, the value for the thermal correction to antioxidant IP (III) was small (-0.20±0.25 kcal/mol, p < 0.05, n = 21) and was not considered. For the TSs, the values of ΔG_TS-R_ and ν_i_ obtained at level II were used to calculate the values of ΔE_COR_ and k(T) at level IV.

Statistical analyses were performed using Social Science Statistics online resource [84]. Illustrations were prepared with the help of MaSK 1.3.0. [85] and VMD [86]. The lipophilicity (logP) of compounds was calculated using the Molinspiration server [87].

## Supporting Information

S1 Table BDE for antioxidants calculated with HF(6-31G(d)), HCTH/407(6-31G(d)), and B3LYP(6-311G(d,p) Gaussian 98) (PDF)

S2 Table Pearson correlation coefficients (R) for the energy values of antioxidants calculated at levels I–IV. Left part: the correlation between energy values (methods I-IV); n = 16. Right part: the correlation between the gas-water differences of the energy values (method IV); n = 16 (24). Before brackets: R for the compounds with H-atom dissociation; in brackets: R for the all compounds. Bold: the values which are not statistically significant (p > 0.05).
(PDF)

S1 Fig The lowest unoccupied molecular orbitals (LUMOs) of kynurenines and phenolic antioxidants. Color scheme, atoms: H–white, C–grey, O–red, N–blue. Isosurface value: 0.05.
(TIFF)

S2 Fig Spin-orbits of kynurenines and phenolic antioxidants after electron abstraction. Color scheme, atoms: H–white, C–grey, O–red, N–blue. Isosurface value: 0.005.
(TIFF)

S3 Fig Spin-orbits of kynurenines and phenolic antioxidants after H-atom abstraction. Color scheme, atoms: H–white, C–grey, O–red, N–blue. Isosurface value: 0.005. Radical O* atom is shown by asterisk.
(TIFF)

S4 Fig The intrinsic reaction coordinates (IRC) for 3HAA–Met-OO* and XAA–Met-OO* complexes. IRC were calculated using B3LYP (level II), for each IRC point the single point energy was calculated using XYG3 (level V). ΔE_TS-X_ is the difference between E_TS_ for saddle-point and E_X_ for the given IRC coordinate. Pearson correlation coefficients R (B3LYP(II)–XYG3(V)) is 0.986 for 3HAA complex (TS area), 0.962 for 3HAA complex (the whole IRC), and 0.918 for XAA complex (the whole IRC).
(TIFF)

S5 Fig J^2^ (Eq 6) as a function of the range-separation parameter μ for different antioxidants (LC-BLYP/6-311G(d,p). (TIFF)

S1 Dataset The optimized structures of antioxidants, radical dimers, and radical adducts. Atom names, nucleus charges, and coordinates (x, y, z; in Å) are given.
(TXT)
