# Supplementary material for: Antioxidant Properties of Kynurenines: Density Functional Theory Calculations
Source: PLoS Comput Biol. 2016 Nov 18;12(11):e1005213. doi: 10.1371/journal.pcbi.1005213 (PMC5115656; doi:10.1371/journal.pcbi.1005213)
Supplement: S1 Table — (PDF) [file pcbi.1005213.s001.pdf]

	I: HF(6-31G(d)); Firefly, full geometry optimization		HCTH/407(6-31G(d)); Firefly, full geometry optimization		III: B3LYP(6-31G(d)); Firefly, full geometry optimization; GAUSSIAN 98, single point energy
**Compound**	**BDE**	**BDE_{COR}**	**BDE**	**BDE_{COR}**	**BDE**
**Water**	81.726	74.197	118.422	111.695	119.707
**Methane**	86.563	77.721	111.922	103.934	111.592
**Phenol**	76.807	69.595	84.702	77.907	93.129
**$L\text{-}3HOK_{NH3+}$**	86.999	79.756	88.219	81.022	93.988
**ASC**	66.929	58.966	81.198	74.437	86.898
**DIBP**	75.692	68.451	79.167	72.446	84.780
**DIBA**	76.137	68.864	77.806	71.118	83.382
**XAA_{OXO}**	81.029	73.758	75.200	68.698	81.419
**DTBP**	71.778	64.247	75.859	69.043	81.635
**DTBA**	72.245	64.726	74.531	67.702	80.121
**L-3HOK**	79.417	71.617	71.146	64.882	77.301
**$2\text{-}NH_2\text{-}Phenol$**	49.668	41.262	71.605	65.041	77.676
**D-3HOK**	78.354	70.012	71.150	64.871	77.310
**3HAA**	78.565	70.887	71.111	64.801	77.312
**$XAA_{OXO/CO2-}$**	76.806	69.556	68.181	61.555	75.749
**DXAN**	43.710	34.146	60.859	54.827	68.312
**$3HAA_{CO2--}$**	43.952	35.971	60.860	54.282	67.097

All values are in kcal/mol.

1. Luo et al., 2007 (Comprehensive handbook...)
2. Amorati et al., 2011.
3. Brigati et al., 2002
4. Borges dos Santos et al., 1998.

-0.092+/- 0.03; N=16 (Firefly-Gaussian)
