# Supplementary material for: Antioxidant Properties of Kynurenines: Density Functional Theory Calculations
Source: PLoS Comput Biol. 2016 Nov 18;12(11):e1005213. doi: 10.1371/journal.pcbi.1005213 (PMC5115656; doi:10.1371/journal.pcbi.1005213)
Supplement: S2 Table — Left part: the correlation between energy values (methods I-IV); n = 16. Right part: the correlation between the gas-water differences of the energy values (method IV); n = 16 (24). Before brackets: R for the compounds with H-atom dissociation; in brackets: R for the all compounds. Bold: the values which are not statistically significant (p > 0.05). (PDF) [file pcbi.1005213.s002.pdf]

	**II**	**III**	**III (LC-BLYP)**	**IV gas**	**IV water**
**E_{HOMO} - BDE**	-0.744	-0.731		-0.532	-0.698
**E_{LUMO} - BDE**	**0.173**	**0.033**		**-0.213**	**0.169**
**H-L gap – BDE**	-0.801	-0.775		-0.663	-0.533
**E_{HOMO} – BDEcor**	-0.738	-0.727			
**E_{LUMO} – BDEcor**	**0.169**	**0.032**			
**H-L gap – BDEcor**	-0.792	-0.768			
**IP - BDE**				0.615	0.793
**E_{HOMO} - IP**			-0.881	-0.989 (-0.985)	-0.901 (-0.884)
**E_{LUMO} - IP**			**-0.449**	-0.844 (-0.867)	**-0.276 (-0.341)**
**H-L gap – IP**			**0.207**	**-0.295 (-0.301)**	**-0.211 (-0.262)**

**IV Δ (gas – water)**	**R**
ΔBDE - ΔIP	0.871
ΔBDE - ΔE_{HOMO}	-0.874
ΔBDE - ΔE_{LUMO}	-0.852
ΔBDE - ΔH-L gap	**-0.407**
ΔIP - ΔE_{HOMO}	-0.992 (-0.991)
ΔIP - ΔE_{LUMO}	-0.984 (-0.988)
ΔIP - ΔH-L gap	**-0.389** (-0.552)
