# Supplementary figures and images for: Antioxidant Properties of Kynurenines: Density Functional Theory Calculations
Source: PLoS Comput Biol. 2016 Nov 18;12(11):e1005213. doi: 10.1371/journal.pcbi.1005213 (PMC5115656; doi:10.1371/journal.pcbi.1005213)

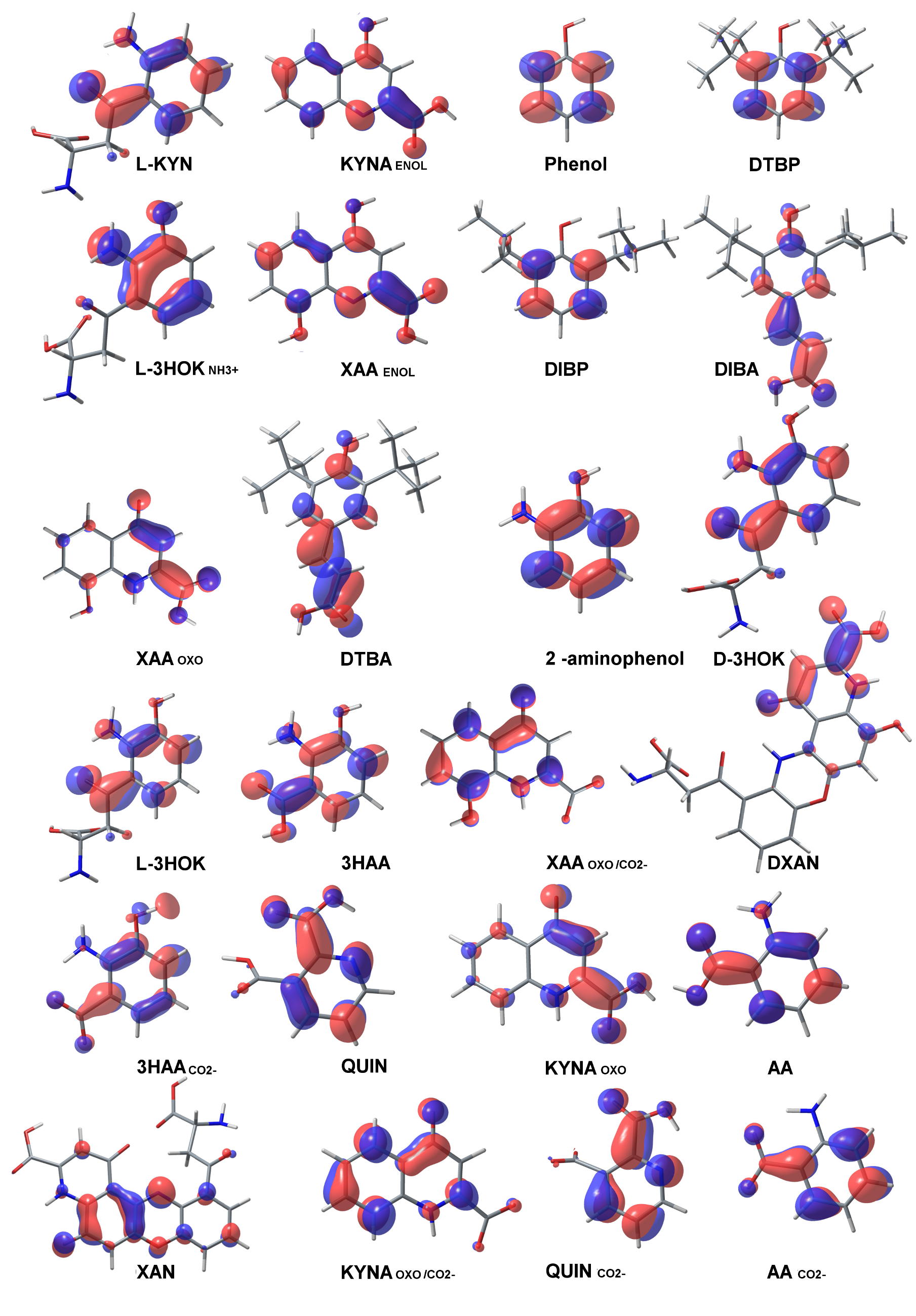

Supplement: S1 Fig — Color scheme, atoms: H–white, C–grey, O–red, N–blue. Isosurface value: 0.05. (TIFF) [file pcbi.1005213.s003.tiff]

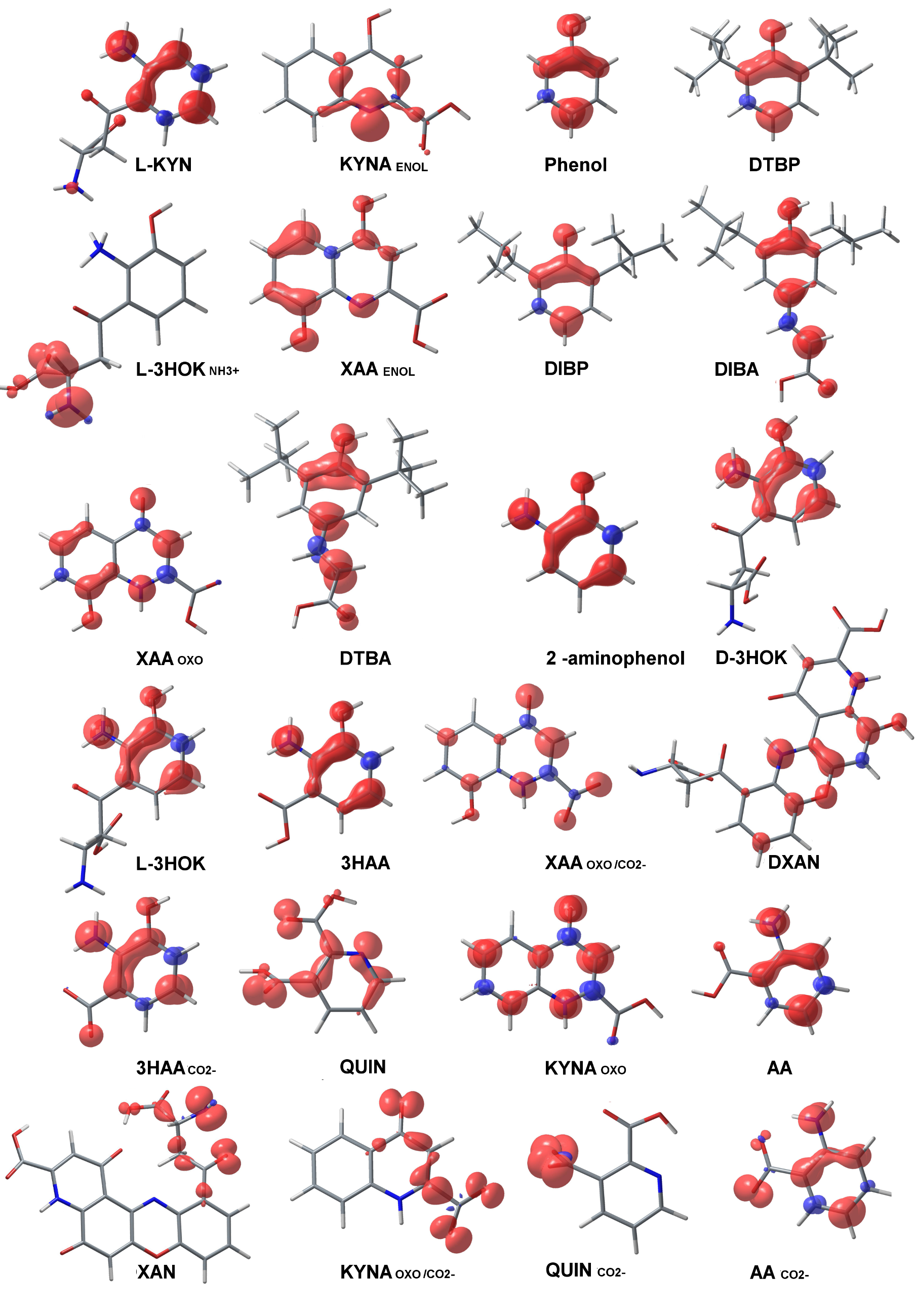

Supplement: S2 Fig — Color scheme, atoms: H–white, C–grey, O–red, N–blue. Isosurface value: 0.005. (TIFF) [file pcbi.1005213.s004.tiff]

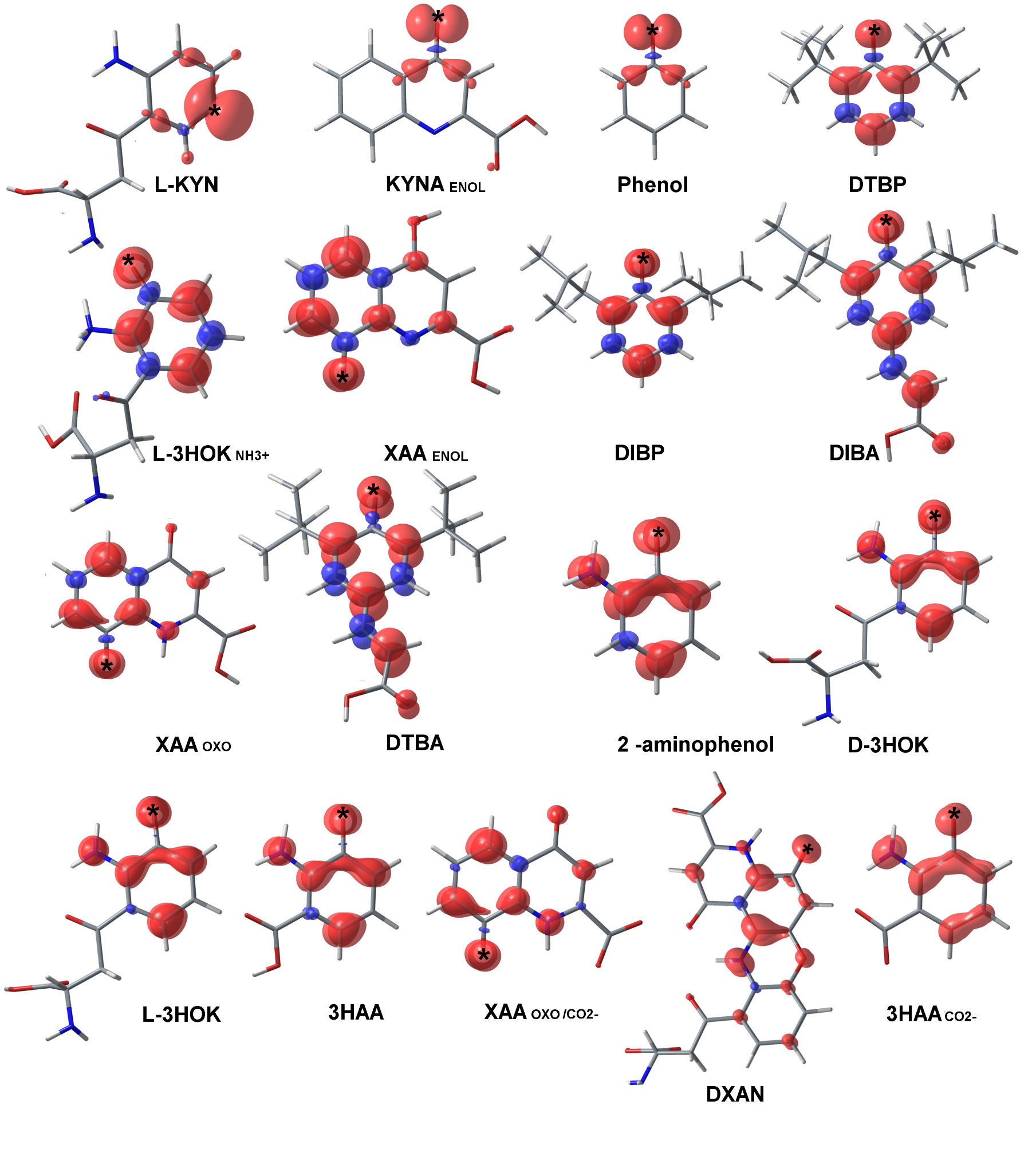

Supplement: S3 Fig — Color scheme, atoms: H–white, C–grey, O–red, N–blue. Isosurface value: 0.005. Radical O* atom is shown by asterisk. (TIFF) [file pcbi.1005213.s005.tiff]

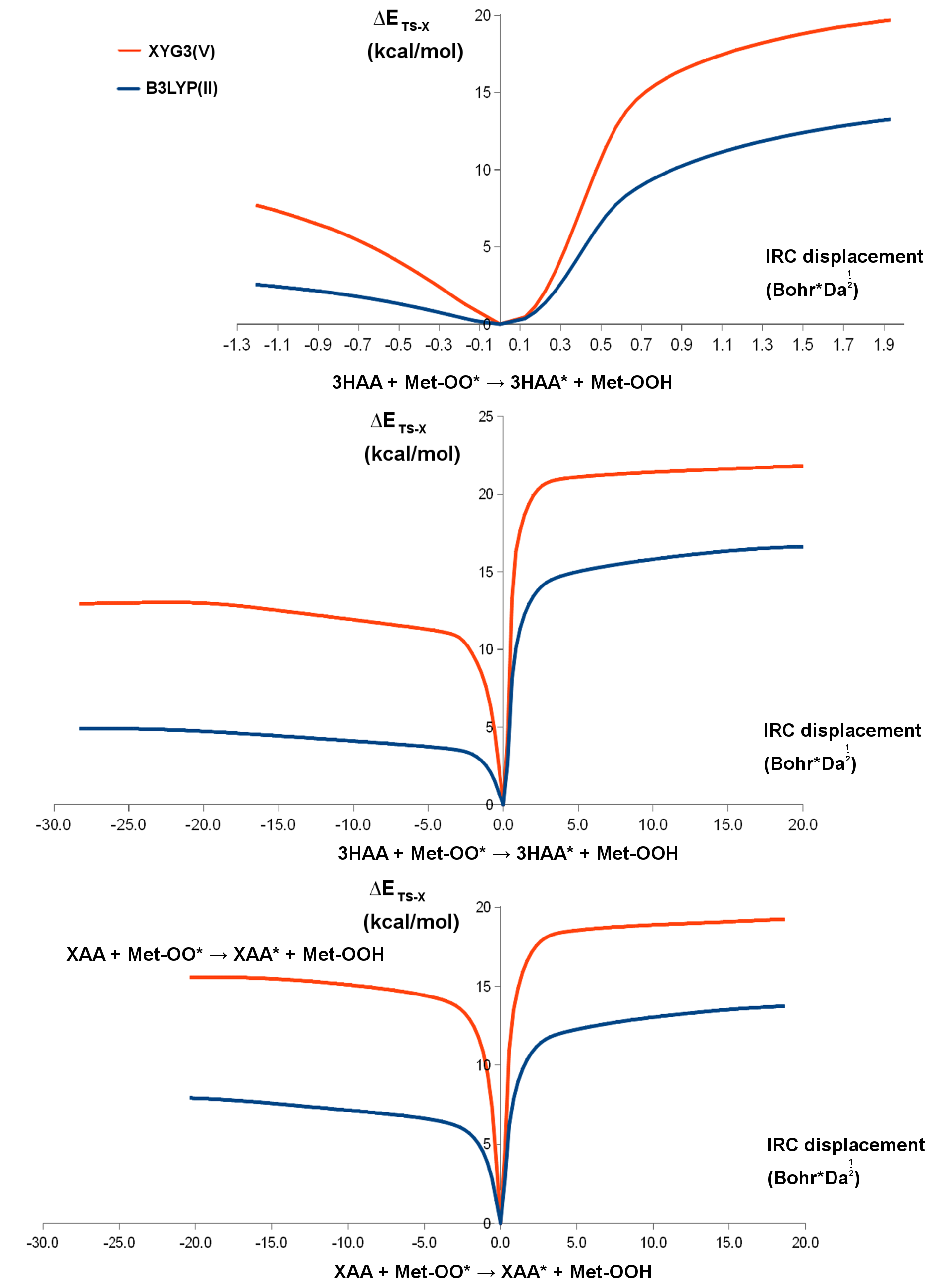

Supplement: S4 Fig — IRC were calculated using B3LYP (level II), for each IRC point the single point energy was calculated using XYG3 (level V). ΔETS-X is the difference between ETS for saddle-point and EX for the given IRC coordinate. Pearson correlation coefficients R (B3LYP(II)–XYG3(V)) is 0.986 for 3HAA complex (TS area), 0.962 for 3HAA complex (the whole IRC), and 0.918 for XAA complex (the whole IRC). (TIFF) [file pcbi.1005213.s006.tiff]

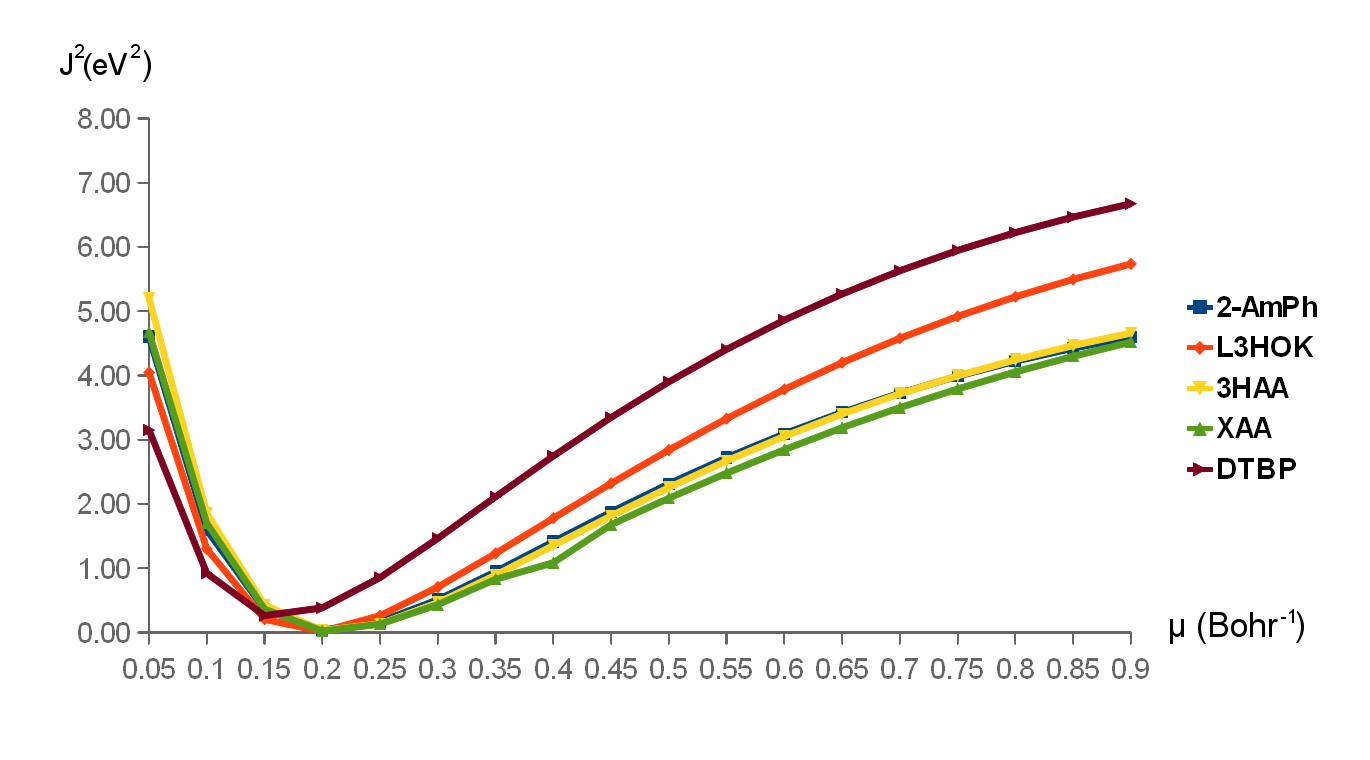

Supplement: S5 Fig — (TIFF) [file pcbi.1005213.s007.tiff]
